# Characterizing approaches used to display antimicrobial resistance data in veterinary and human medicine: a scoping review

**DOI:** 10.1017/ash.2025.10243

**Published:** 2025-12-17

**Authors:** Famke Alberts, Leilani Rocha, Emily He, Sheila Keay, Kurtis Sobkowich, Casey L. Cazer, Scott Weese, Theresa Bernardo, Zvonimir Poljak

**Affiliations:** 1 Department of Population Medicine, Ontario Veterinary College, University of Guelph, Guelph, ON, Canada; 2 Department of Clinical Sciences, College of Veterinary Medicine, Cornell University, Ithaca, NY, USA; 3 Department of Public and Ecosystem Health, College of Veterinary Medicine, Cornell University, Ithaca, NY, USA; 4 Department of Pathobiology, Ontario Veterinary College, University of Guelph, Guelph, ON, Canada

## Abstract

**Introduction:**

Antimicrobial resistance (AMR) is a complex One Health problem that requires continuous surveillance to minimize the potential hazards. Information must be disseminated promptly in easily understandable formats to support informed decisions and actions by data end-users. One way to address this is through real-time visualizations, such as dashboards, to help key interest-holders understand and monitor AMR. A scoping review was conducted to understand the current body of evidence surrounding real-time AMR visualizations in both veterinary and human health.

**Methods:**

Twelve sources were searched for relevant citations. 1763 citations were included in the screening process. Citations were screened for four main criteria: (i) the text had to be a primary research article in English (ii) published between 1990 and 2023, and (iii) it had to discuss the methodology of an AMR display (iv) that was updated at least quarterly.

**Results:**

Forty-two publications were identified as relevant. Publication information, information about the data used in the described displays, display information, and user information were charted. Publications were from 25 countries and utilized data from over 40 databases. Various bacterial genera and species were reported; the most common bacterial species were *Escherichia coli* and *Staphylococcus aureus*. Displays were most focused mainly on human data.

**Conclusions:**

AMR data visualization has been implemented globally and is a critical component of continued AMR surveillance. Displays are often part of a larger surveillance system. A key challenge is designing a visualization for an intended audience and the information then being utilized by that audience.

## Introduction

Antimicrobial resistance (AMR) is a significant health challenge for human and animal populations and is a complex One Health problem.^1^ AMR is the ability of a microorganism to be resistant or tolerant to chemotherapeutic agents, antimicrobial agents, or antibiotics.^2^ AMR occurs naturally over time with the change in the genetic composition of microorganisms and the use of antibiotics as a treatment.^1,3^

Continuous monitoring of AMR is necessary to minimize and understand its effects.^4^ To implement an effective surveillance system there must be collection, analysis, interpretation, and timely circulation of antimicrobial susceptibility testing results (AST) and AMR data.^5^ A dashboard system and adjacent dashboard-like displays provide the opportunity to assist in implementing an effective surveillance system.^6^ They can be utilized for ongoing data sharing and can be an effective visualization capturing the dynamic nature of public health concerns.^7^ These real-time systems can be implemented to support multiple interest-holders, including clinicians, policymakers, and researchers.^7^

Time is a significant barrier for medical practitioners^8^ and veterinarians^9^ to stay current with research. This raises concerns since research synthesis under the framework of evidence-based medicine requires time and robust steps to mitigate bias.^10^ When faced with urgent decision-making, access to user-friendly, accurate, and interpretable displays of AMR data may be beneficial. Visualization can provoke necessary questions and aid in AMR monitoring.^11^ These premises form the focus of this review.

Available information characterizing and comparing existing approaches to real-time displays of human and veterinary AMR data may be limited. Safdari et al. (2020) published a scoping review of publications current to 2016 comparing computational methods and algorithms used in AMR surveillance systems to aid in human healthcare and policy decision-making.^12^ The current review will expand its purpose to include veterinary AMR data and all approaches to data display systems, including non-computational methods. Baede et al. (2022) and Diallo et al. (2020) also published reviews on current AMR surveillance systems.^13,14^ However, these did not focus on AMR displays. Review findings may be used as a basis for further investigation of the nature and purpose of end-user access to AMR data display systems, evaluation of the effectiveness of AMR display systems for improving decision-making, and further development of optimal interactive displays. This scoping review addresses the research question: “What approaches are used to display antimicrobial resistance data in real-time for surveillance or clinical decision-making?”

## Materials and methods

### Protocol and registration

The protocol for this review titled “Characterizing Approaches Used to Display Antimicrobial Resistance Data in Veterinary and Human Medicine: A Scoping Review Protocol” was prepared using the Preferred Reporting Items for Systematic Reviews and Meta-Analyses Extension for Scoping Reviews (PRISMA-ScR) reporting guidelines.^15^ The protocol was posted on the University of Guelph Institutional Repository (The Atrium, https://hdl.handle.net/10214/28406) on July 7th, 2024 (S1 Text).

After the publication of the protocol, the following modifications were made:

- The inclusion criterium for the publication year was extended to 2023 because an updated search was performed on October 17^th^, 2023.
- The study deduplication process was additionally performed using Distiller-SR (© 2023 Systematic Review Software by Evidence Partners).^16^
- Data were not charted for the following:
  - AMR data database affiliation.
  - Additional demographic information displayed in the AMR displays beyond temporal and geographic.
  - If a defined protocol was established for each display.

### Eligibility criteria

To be eligible for inclusion, publications had to be full-text English language publications published between January 1990 and October 17^th^, 2023. 1990 was selected to align with the earliest identified publication in Safdari et al. (2020) which was used as a reference because of the similar search parameters.^12^ Publications were restricted to primary research articles and conference proceedings that described a methodology for real-time displays of AMR data, where real-time is defined as a time lag of no more than quarter-yearly updates. There were no restrictions regarding the outcomes of data collected and displayed. Gray literature was excluded (aside from conference proceedings). Review articles were excluded but tagged to be used for additional literature search.

### Information sources

On June 7^th^, 2022, and October 17^th^, 2023, five bibliographic databases were searched through four bibliometric platforms (Table S11). Proceedings from four conferences were hand-searched (Table S12), as were references of relevant publications. Safdari et al. (2020), Diallo et al. (2020), and Baede et al. (2022) were hand-searched for relevant references.^12–14^ The ‘snow-balling’ method, as described in Jalali and Wohlin (2012)^17^ was used to search the key AMR surveillance websites: Global Antimicrobial Resistance and Use Surveillance System (GLASS), Canadian Integrated Program for Antimicrobial Resistance Surveillance (CIPARS), IDEXX, The European Antimicrobial Resistance Surveillance Network (EARS-Net), and The National Antimicrobial Resistance Monitoring System (NARMS).^18–22^ See S1 Table for the search strings for all the bibliographic databases.

### Selection of sources of evidence (relevance screening) and data charting process

Citation metadata from the search output was compiled and deduplicated using Mendeley reference management software.^16^ Citation metadata were then uploaded to Distiller-SR (© 2023) software package and deduplicated again. Relevance screening was performed in two stages (Level 1 and 2) using forms built in Distiller-SR (© 2023). Level 1 relevance screening was done using title and abstract. Level 2 screening used full texts. Two reviewers performed screening and data charting, followed by conflict resolution. If an agreement could not be reached, a third reviewer was consulted. The authors of references were not contacted for additional information. Full texts were obtained using EndNote citation management software^23^ and through hand-searching and then uploaded to Distiller-SR (© 2023). The forms used are outlined in S2 Table.

Publication information that was charted was first author country, affiliation, year of publication, and funding information.

Data methodology was charted by the source of the data used in the display; the geographic coverage of the database; source species; bacterial species; antimicrobial and sample restrictions; and how repeat sampling was handled. Restrictions refer to boundaries on the displayed information. For example, a display showing only *Escherichia coli* (*E. coli*) AMR data from urine samples but shows any antimicrobial that is uploaded would be restricted for bacteria (*E. coli*) and sample type (urine) but unrestricted for antimicrobials. Sample type was defined as the biological material where the sample was taken from for culture. Displays were evaluated regarding whether sample type data was displayed and if restricted, the sample types used within the displays were charted (eg, blood, urine). A single patient or sample site may be sampled multiple times during the same illness and may be treated with antimicrobials prior to sampling; both factors can influence AMR analyses. Data were charted to see if publications accounted for either repeat samples or previous treatment and, if these data were accounted for and how..

The type of display was charted as described by the authors resulting in discrepancies in reporting. Display outcomes were categorized as quantitative, qualitative, not stated or as displaying data related to drug resistance genes. Gene resistance displays showed AMR resistance genes through either a database search or analysis of an uploaded sequence. Quantitative outcomes displayed count data and minimum inhibitory concentration (MIC) values. Qualitative outcomes displayed susceptible/intermediate/resistant (SIR) data and other phenotypic data. Display workflows were separated into stages. These stages included the initial user input, storing the inputted data in a database, and then using the data from the database in the final display output. Data were charted for the way the display was updated, which encompassed the transition from database to display. Displays were either automatically updated with information provided in the database or manually updated by an individual. The customizability of the display was defined by a user’s ability to alter what was being displayed through filters. The functionality of the displays was determined using only links or directions provided within the publication. Functionality was based on a functioning display as of May 2024. Lastly, the geographic and temporal data captured within displays was charted. Displays could display multiple levels geographically and temporally.

User methodology was charted as intended users and users with access. The intended users were the goal users of the display, as stated in the publication and the users the display was created for. The users with access are the users that can view the display.

### Synthesis of results

Data were downloaded from Distiller-SR (© 2023) into R using RStudio^24,25^ for cleaning, analysis, and creation of relevant Figures and tables.

## Results

**Figure 1.**
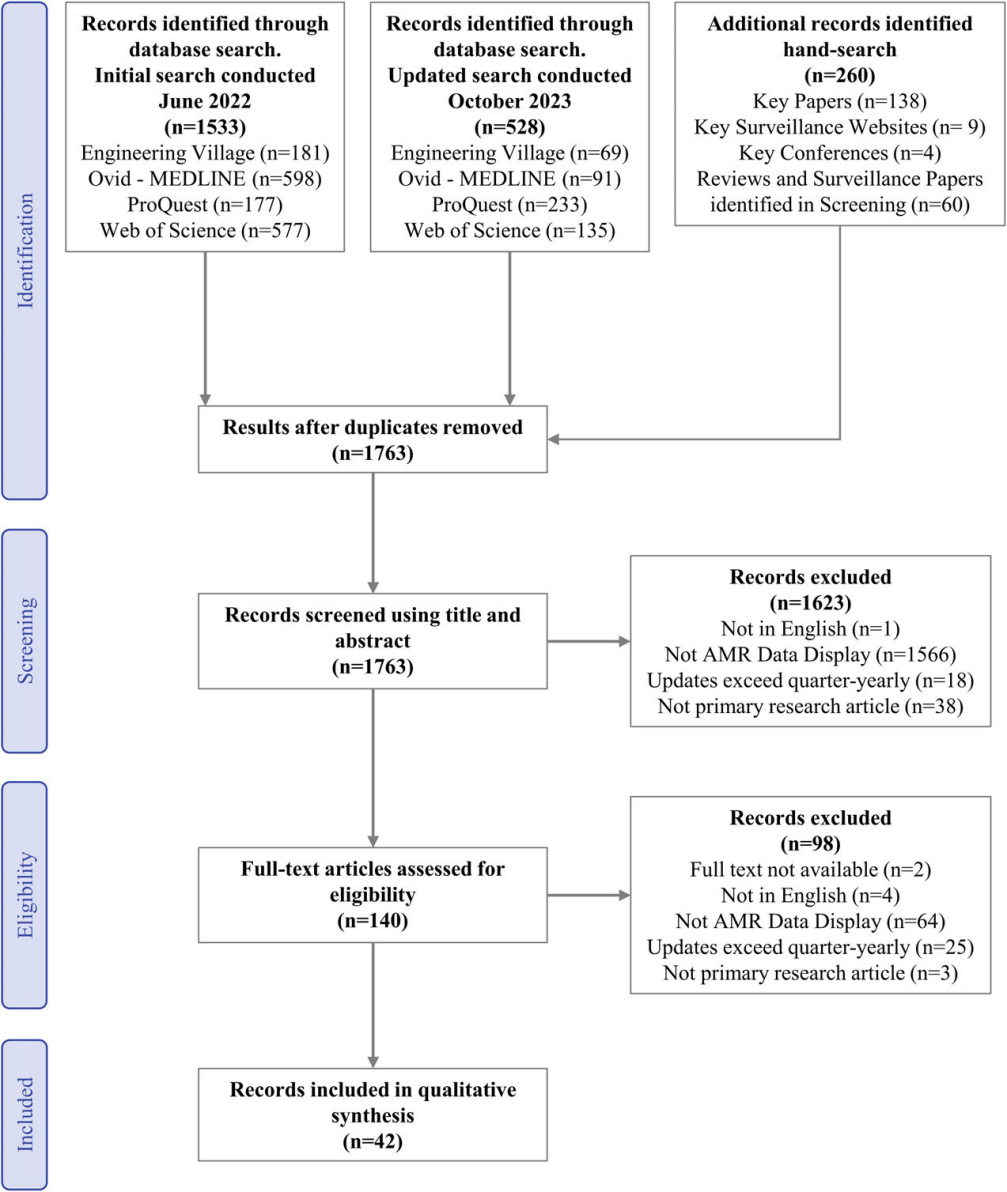
Preferred Reporting Items for Systematic Reviews and Meta-Analyses (PRISMA) flow chart for screening and selection of the publications to be included in qualitative synthesis. Key conferences and surveillance papers are listed in the methods and Table S12.

The search resulted in 1763 citations after deduplication. After screening, 42 publications were included in data charting. The PRISMA flow chart shows the selection process (Figure 1). The 42 included papers and their objectives are in Table S3.

### Publication information

**Figure 2.**
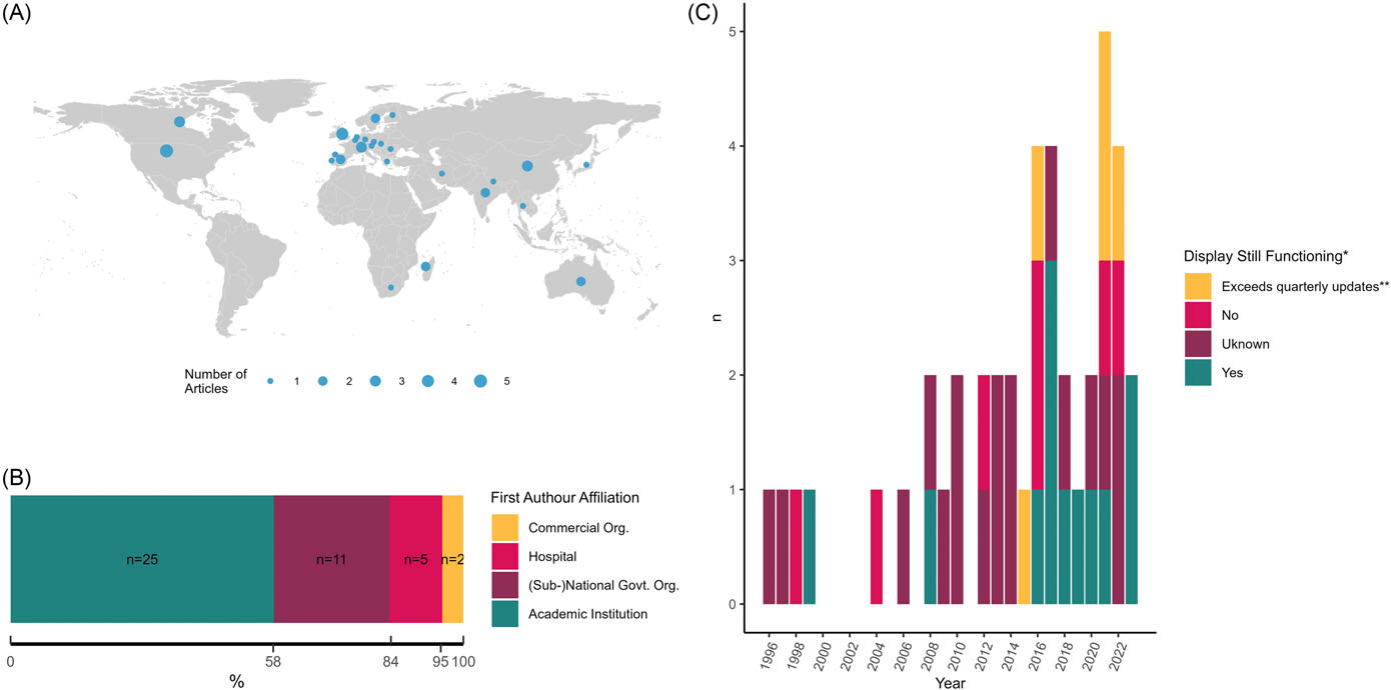
Publication information charted. A. First author country. The country that the first author of selected publications is affiliated with. B. The first author’s organizational affiliation. C. The number of publications by year of publication and functionality of the display. * The functionality is based on the functionality of the display as of May 30, 2024. ** These displays were included after screening because in the publication authors stated the display would be updated more frequently or it was unclear, however, since publication the display is no longer updated more than quarter-yearly.

First authors were affiliated with institutions in 25 countries (Figure 2A). The top five countries were the United States of America (*n* = 5/42 publications, 11.9%), United Kingdom (*n* = 4/42, 9.5%), Canada (*n* = 3/42. 7.1%), China (*n* = 3/42, 7.1%) and Switzerland (*n* = 3/42, 7.1%) (Table 1, Table S4). The first authors were affiliated with academic institutions (*n* = 25/42, 59.5%), government organizations (*n* = 11/42, 26.2%), hospitals (*n* = 5/42, 11.9%) and commercial organizations (*n* = 2/42, 4.8%) (Figure 2B, Table 1). MacFadden et al. (2016) was affiliated with both an academic institution and hospital.^26^ The oldest publication was published in 1996,^27^ and the most recent were published in 2023.^28,29^ The number of publications is consistent throughout the years with a slight spike (*n* = 5/42, 11.6%) in 2020 (Figure 2C). Funding information is provided in Table S3.

**Table 1.** General characteristics of publication information for all selected publications

Publication characteristic	Number of publications (*n* = 42)†	Percentage (%)
**First author country:**		
United States of America	5	11.9
United Kingdom	4	9.5
Canada	3	7.1
China	3	7.1
Switzerland	3	7.1
Other‡	24	57.1
**First author institutional affiliation:**		
University	25	59.5
(Sub-)National Government Organization	11	26.2
Hospital	5	11.9
Commercial organization	2	4.8

† Publications may belong to multiple categories

‡ S4 Table includes full list of countries.

### Data methodology

Table 2 provides an overview of data methodology charted. The source of the data used in the display was collected for all 42 publications. In 19 publications, the data source used was not specified (*n* = 19/42, 45.2 %). The most common data sources were PubMed (*n* = 5/42, 11.9%), Gen Bank (*n* = 4/42, 9.5%) and NCBI (*n* = 2/42, 4.8%) (Table 2). Thirty-six other data sources were also used and displays could use multiple data sources (Table S5). The geographic level of coverage for the databases were international (*n* = 15/42, 35.7%), site-specific (*n* = 12/42, 28.6%), national (*n* = 9/42, 21.4%), regional (*n* = 2/42, 4.8%), and supra-national (*n* = 1/42, 2.4%), and for some was not stated (*n* = 3/42, 7.1%) (Table 2).

**Table 2.** General characteristics of data used in the displays for all selected publications

Data characteristic	Number of publications (*n* = 42)†	Percentage (%)
**Database name:**		
Unspecific name(s)	19	45.2
Pubmed	5	11.9
GenBank	4	9.5
NCBI	2	4.8
Other‡	36	85.7
**Database geographic level of coverage:**		
International	15	35.7
Supra-National	1	2.4
National	9	21.4
Regional	2	4.8
Site-specific	12	28.6
Not stated	3	7.1
**Source species:**		
Human	33	78.6
Animal	10	23.8
→ Livestock	→ 4	9.5
► Avian, swine, bovine, ovine	►1	2.4
→ No restrictions	→ 3	7.1
→ Not stated	→ 3	7.1
Not stated	7	16.7
**Bacteria:**		
Genera-specific	5	11.9
*→ Enterococcus*	→ 2	4.8
*→ Escherichia*	→ 2	4.8
*→ Salmonella*	→ 2	4.8
*→ Staphylococcus*	→ 2	4.8
→ Other‡	→ 8	19.0
Species-specific	12	28.6
*→ Escherichia coli*	→ 7	16.7
*→ Staphylococcus aureus*	→ 7	16.7
*→ Klebsiella pneumoniae*	→ 4	9.5
*→ Pseudomonas aeruginosa*	→ 4	9.5
*→ Streptococcus pneumoniae*	→ 4	9.5
→ Other‡	→ 20	47.6
No restrictions	13	31.0
Not stated	16	38.1
**Antimicrobial restrictions:**		
No restrictions	14	33.3
Yes restrictions	11	26.2
Not stated	17	40.5
**Sample type data used:**		
Yes	23	54.8
*Sample types restricted*		
→ Yes	→ 8	19.0
*Sample types*		
► Blood	►5	11.9
► Urine	►5	11.9
► Respiratory	►3	7.1
► Other‡	►7	16.7
► Not stated	►1	2.4
→ No	→ 8	19.0
→ Not stated	→ 7	16.7
No	8	19.0
Not stated	11	26.2
**Repeat sample or previous treatment:**		
Repeat sample/results excluded	6	14.3
Human verification of repeats	4	9.5
Excluded uncorrected errors in data	2	4.8
Merge data from same patient	1	2.4
Quality checking	1	2.4
Readmission alerts for previous treatment.	1	2.4
Require statement	1	2.4
Not stated	32	76.2

† Some publications may belong to multiple categories

‡ S5 Table includes full list of database names, S6 Table includes full list of bacteria genera, S7 Table includes full list of bacteria species, S8 Table includes full list of sample types.

Twenty-five displays focused on only human data (*n* = 25/42, 60%); however, some focused-on animals (*n* = 2/42, 5%) or both humans and animals (*n* = 8/42, 19%). Seven publications did not state which source species was used (*n* = 7/42 publications, 16.7%) (Table 2). The ten publications displaying animal data were categorized into specific categories. Four fell in the category of livestock data (*n* = 4/10 publications using animal data, 40%), three displays did not have restrictions on the animals (*n* = 3/10, 30%) and three did not state which animal data were displayed (*n* = 3/10, 30%). Of the four publications that used livestock, one specified that it used avian, swine, bovine, and ovine as their source species (*n* = 1/4 publications that used livestock, 25%).

Bacteria were charted at either the genus level (*n* = 5/42, 11.9%) or species level (*n* = 12/42, 28.6%). Additionally, some publications did not state which bacteria were used within their display (*n* = 16/42, 38.1%), thirteen displays had no restrictions on the bacteria displayed (*n* = 13/42, 31.0%) (Table 2). Most displays that stated which bacteria were included utilized multiple types of bacteria (*n* = 22/26 publications with bacteria stated, 84.6%). Of the five publications that specified the level of genus, the most common were *Enterococcus* (*n* = 2/5 publications that used genus, 40%), *Escherichia* (*n* = 2/5, 40%), *Salmonella* (*n* = 2/5, 40%), and *Staphylococcus* (*n* = 2/5, 40%) (Table 2, Table S6). Of the publications that reported genus level data, an average of 3.2 genera were reported per publication (minimum 1, maximum 6, median 4). Twelve publications specified to the species level. The most common species were *E. coli* (*n* = 7/42, 16.7%), *S. aureus* (7/42, 16.7%), *Klebsiella pneumoniae* (*n* = 4/42, 9.5%), *Pseudomonas aeruginosa* (*n* = 4/42, 9.5%), and *Streptococcus pneumoniae* (*n* = 4/42, 9.5%) (Table 2, Table S7). Of the 12 publications that specified species, on average, 13.5 species were used per publication (minimum 1, maximum 116, median 3.5).

In 14 publications, there were no restrictions on the antimicrobials that were displayed (*n* = 14/42, 33.3%). In 11, there were antimicrobial restrictions (*n* = 11/42, 26.2%), and in 17 publications, it was not stated if there were restrictions (*n* = 17/42, 40.5%) (Table 2). Of the 11 publications in which it was specified that there were antimicrobial restrictions two did not state how many antimicrobials were used. The other nine publications displayed on average, 20.6 antimicrobials (minimum 1, maximum 55, median 15).

Sample type data were used in 23 publications (*n* = 23/42, 54.8%, and in *n* = 4/10 publications which displayed animal information [40%]), not used in eight publications (*n* = 8/42, 19.0%, *n* = 1/10, 10%), and not stated in 11 publications (*n* = 11/42, 26.2%, *n* = 5/10, 50%). Of the 23 publications in which sample type data were used, sample types were restricted in eight (*n* = 8/23 publications using sample type, 34.8%), sample types were not restricted in eight (*n* = 8/23, 34.8%, *n* = 2/4, 50%), and it was not stated if sample type was restricted in seven (*n* = 7/23, 30.4%, *n* = 2/4, 50%). Of the eight publications that did restrict sample type, the most common sample types were blood (*n* = 5/8 publications that restricted sample type, 62.5%), urine (*n* = 5/8, 62.5%), and respiratory (*n* = 3/8, 37.5%) (Table 2, Table S8). Kaur et al. (2021) did not specify the sample types utilized; however, the display was restricted to 80 different human sample types.^30^ Of the eight publications that did restrict sample type, authors used an average of 12.4 sample types (min 1, max 80, med 2.5).

Commonly, authors did not state how repeat samples or previous treatment data were handled (*n* = 32/42 publications, 76.2%). Among the remaining 10 displays: (i) repeat samples/results were excluded (*n* = 6/10 publications handling repeat data, 60%), (ii) there was human verification of repeated samples (*n* = 4/10, 40%), or (iii) there was some form of check or statement performed (Table 2). Human verification of repeated samples involved flagging data that previously appeared or seemed erroneous in some other capacity would be flagged and then a human would review the data before adding it to the display.

### Display information

Table 3 provides an overview of the display information charted. Most displays were reported as web applications (*n* = 15/42 publications, 25.7%), dashboards (*n* = 6/42, 14.3%), database systems (*n* = 6/42, 14.3%), and reports (*n* = 6/42, 14.3%) (Table 3). In two publications, the displays were stated as both reports and alerts and were charted in both categories.^31,32^ Most of the web applications were publicly accessible displays (*n* = 9/15, 60%). All 6 of database systems were publicly accessible displays. Five publications (*n* = 5/42, 11.9%) reported their display as a decision support system, the intended users for these five displays were authorized users in a hospitals.

**Table 3.** General characteristics of display charted from the selected publications

Display characteristic	Number of publications (*n* = 42)†	Percentage (%)
**Type of display**		
Web application or web tool	15	35.7
Dashboard	6	14.3
Database system	6	14.3
Decision support system	5	11.9
Reports	4	9.5
Information system	3	7.1
Reports and alerts	2	4.8
Alerts	1	2.4
**Outcomes displayed:**		
Gene resistance	10	23.8
Quantitative	23	54.8
Qualitative (SIR, phenotypes etc.)	18	42.9
Not stated	6	14.3
**Display update method:**		
Automatic	28	66.7
Manual	8	19.0
Not stated	6	14.3
**Customizable by user:**		
Yes	31	73.8
No	6	14.3
Not stated	5	11.9
**Functionality of the display§:**		
Functioning	17	40.5
→ Updates now exceed quarter-yearly	→ 5	11.9
Not functioning	7	16.7
Unknown	18	42.9
**Geographic coverage:**		
Exact GPS location	3	7.1
Primary care organization	20	47.6
Region	12	28.6
State/province	2	4.8
Country	9	21.4
Other	4	9.5
Not stated/not displayed	11	26.2
**Temporal display**		
Real-time	4	9.5
Day	6	14.3
Week	3	7.1
Month	12	28.6
Quarter	4	9.5
Year	17	40.5
Not stated/not applicable	14	33.3
**Intended users‡:**		
Health professionals	33	78.6
Researchers	28	66.7
Policy makers	17	40.5
Public	7	16.7
Not stated	2	4.8
**Users with access:**		
Public	20	47.6
Authorized users	18	42.9
Not stated	4	9.5

† Publications may fit in multiple categories.

‡ Complete list in Table S9§ Functioning as of May 30th, 2024.

Display outcomes were categorized as gene resistance, quantitative, qualitative, and not stated. Fifteen displays had multiple outcome types (*n* = 15/42, 35.7%). There were 23 publications in which quantitative outcomes were displayed (*n* = 23/42, 54.8%), 18 qualitative (*n* = 18/42, 42.9%), ten gene resistance (*n* = 10/42, 23.8%), and six did not state the outcome being displayed (*n* = 6/42, 14.3%) (Table 3). Of the 10 displays for gene resistance, 8 were accessible at a public level (*n* = 8/10 gene resistance displays, [80%]).

Most of the displays were automatically updated (*n* = 28/42, 66.7%), eight were updated manually (*n* = 8/42, 19.0%), and in six publications, it was not stated how the display was updated (*n* = 6/42, 14.3%) (Table 3).

For most of the displays, the users were able to customize the display themselves in some way (*n* = 31/42, 73.8%). Six displays were not customizable by the user (*n* = 6/42, 14.3%), and in five publications, it was not stated if the display was customizable (*n* = 5/42, 11.9%) (Table 3).

Forty percent of the displays in the publications were still functioning (*n* = 17/41, 40.5%). However, five of the displays were no longer updated, or the updates exceeded quarter-yearly, which was the criterion for a frequently updated display. Seven of the displays were no longer functioning (*n* = 7/42, 16.7%). It was unknown whether the remaining displays were still functioning (*n* = 18/42, 42.8%). These were usually private displays (e.g., part of a hospital data system), or there was no stated access point within the publication (Table 3). The oldest publication with a display that was still functioning was Vatopoulos et al. (1999) and the second oldest was Liu and Pop (2009).^33,34^ All other displays that were still functioning, including those exceeding quarterly updates, were published within the last ten years (2015–2023).

Geographic information was captured from most to least granular and categories were not mutually exclusive. These levels were at the exact GPS location (*n* = 3/42, 7.1%), primary care organization (*n* = 20/42, 47.6%), region (*n* = 12/42, 28.6%), state/province (*n* = 2/42, 4.8%), country (*n* = 9/42, 21.4%), and other levels (*n* = 4/42, 9.5%). This information was either not displayed or not stated in 11 publications (*n* = 11/42, 26.2%). Of the 20 publications that displayed primary care information geographically, 13 of these were displays intended for hospitals (*n* = 13/20, 65%). Temporal information was charted similarly from most to least granular and categories were not mutually exclusive. Temporal information was charted as real-time (*n* = 4/42, 9.5%), day (*n* = 6/42, 14.3%), week (*n* = 3/42, 7.1%), month (*n* = 12/42, 28.6%), quarter (*n* = 4/42, 9.5%), year (*n* = 17/42, 40.5%). Temporal information was either not stated or not displayed in 14 (*n* = 14/42, 33.3%) publications (Table 3).

### Users

**Figure 3.**
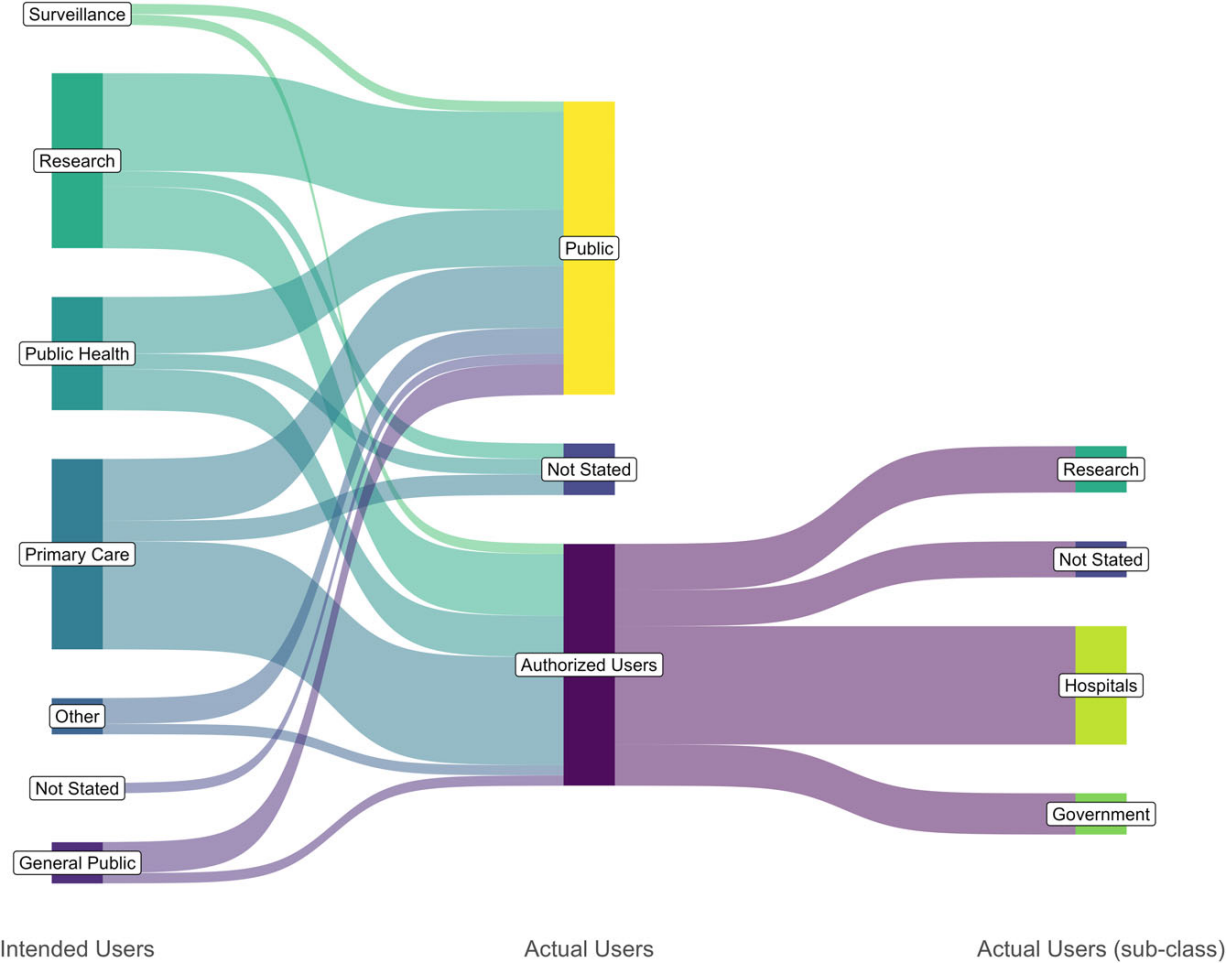
Sanky diagram for the intended users of the data displays versus the users with actual access. Full list of the intended users is available in S9 Table. The intended users of the data displays was charted from the publications as the users that the authors had made the display for. The actual users are who has access to the display, the authorized users section is further broken down into sub-classes.

The users of displays are shown in Table 3 and Table S9. The intended users versus those with access are shown in Figure 3. Altorf-van der Kuil et al. (2017) and Li et al. (2020) describe displays with both a public-facing and authorized user interface with different levels of access and visibility for the different user levels.^35,36^

## Discussion

Many displays were at the geographical level of hospital (*n* = 20/42, 47.6%) with the intended user being within a hospital (*n* = 28/42, 66.7%). This focus on hospital care is logical when considering the impact AMR and AST results can have on hospital antimicrobial use decision-making and the importance of up-to-date information, including antibiograms, for adequate patient care. This was reflected by the term decision support system only being used to describe displays created for hospital settings (*n* = 8/42, 19.0%).

In addition to identifying the overall approaches of these displays, four main areas for future research were identified. These include a lack of standardized reporting and reporting of methodology, a lack of epidemiological indicators of quality, the difference in intended user and actual user, and a gap in environmental and animal related real-time visualizations.

When charting data for the selected publications there were many times where: (i) it was unclear what methodology was used to display results, (ii) important contextual information for understanding displays was lacking, and (iii) steps in data processing were not fully described. This becomes evident when looking at the number of “Not Stated” results throughout Table 2 and 3. Description of underlying display methodology is key to being able to interpret display data correctly but also for continued advancements of displays. A framework could be developed for the reporting of health display systems to standardize and provide guidelines when sharing the development of new displays. Barbazza et al., (2022), highlighted the importance of ensuring interpretation of the dashboards was straightforward and providing explanations for all components of a display.^37^

A greater focus on how the epidemiological quality of data has been assessed prior to display should be included to ensure a full understanding of the methodology. In 76.2% (*n* = 32/42) of publications it was not stated how previous treatment data were incorporated into the display and this is only one metric of epidemiological quality assessment. Other metrics include the considerations of culturing practice and accounting for temporal outbreaks (CLSI, 2022).^38^ One issue with previous treatment is the definition of what it is, and can be defined within a display. Another consideration is that treatment data is not always readily available.

Per Figure 3, the intended users of displays and the actual users of displays did not always align. This should be a key focus in display development as the target audience will affect what is being displayed and how it is being displayed. If a display designed for one audience is accessed by another, its effectiveness decreases. If parties that cannot interpret the data being presented are provided access this may cause unintended consequences such as inappropriate antibiotic use. Barbazza et al., (2022), discussed similar themes, that there were often challenges in managing users and user interpretation of the data specifically relaying information in lay terms for a wide public audience.^37^ Additionally, Barbazza et al., (2022) reported, that in the early stages of dashboard development target users were not being explicitly defined.^37^

Incorporating more animal and environmental could be beneficial to a broader understanding and incorporating a One Health approach. Oberin et al., (2022), noted that a challenge for the incorporation of environmental data in these systems is that surveillance of environmental AMR is not well established in many countries.^39^

The information presented could be used by researchers to help inform current gaps in the literature such as methodology reporting. Additionally, this review could be of use to policy makers and health authorities to help inform them on how other authorities have reported information and selected parameters such as pathogens and sample type.

Limitations of this scoping review include that only peer-reviewed literature was searched. However, more displays likely exist in gray literature, and are likely not reported on publicly, especially if part of a healthcare system or integrated into a hospital setting. Glowney et al., (2023), are conducting a scoping review regarding multidrug resistance visualizations that included gray literature via a Google search.^40^ Lastly, the publications were limited to the English language.

## Conclusion

There were many approaches to displaying AMR data. The way a display was created and what was included were heavily reliant on the use case. Most displays were created with the purpose of being used within a hospital setting. As information systems and integrated real-time displays continue to evolve so must the methodology for reporting these displays. Better reporting standards and practices will help displays become more accessible, more understandable and will contribute to data sharing within the scientific community.

## Supporting information

10.1017/ash.2025.10243.sm001 Alberts et al. supplementary material Alberts et al. supplementary material

## Supplementary material

The supplementary material for this article can be found at https://doi.org/10.1017/ash.2025.10243.

## Research transparency and reproducibility

The protocol for this scoping review is posted on the university of Guelph Atrium (https://hdl.handle.net/10214/28406) additional data can be found in the supplementary materials.
