# Supplementary material for: Characterizing approaches used to display antimicrobial resistance data in veterinary and human medicine: a scoping review
Source: Antimicrob Steward Healthc Epidemiol. 2025 Dec 17;5(1):e344. doi: 10.1017/ash.2025.10243 (PMC12722559; doi:10.1017/ash.2025.10243)
Supplement: Alberts et al. supplementary material [file S2732494X2510243Xsup001.zip › S1 Table.docx]

S1 Table: The search strings used during database search for the scoping review.

| **Clarivate, Web of Science Core Collection – Date Range 1990-2023** |
| --- |
| ((TI=((ant*microb* OR ant*biot* OR ant*bacter*) AND (resistance OR susceptibility))) OR (AU=((ant*microb* OR ant*biot* OR ant*bacter*) AND (resistance OR susceptibility)))) AND (TS=((dashboard OR application OR "web-based tool" OR "web based tool" OR interactive OR visuali?ation OR display OR decision support OR decision-support OR information system OR user interface design OR user-interface design) AND (surveillance OR database OR updat* OR real-time OR real time))) |
| **Elsevier, Engineering Village- Inspec and Compendex** |
| (((((((ant*microb* OR ant*biot* OR ant*bacter*) AND (resistance OR susceptibility)) AND (dashboard OR application OR "web based tool" OR "web-based tool" OR interactive OR visuali?ation OR display OR decision support OR decision-support OR "information system" OR user interface design OR user-interface design) AND (surveillance OR database OR updat* OR real-time OR "real time")) WN KY) AND ((cpx OR ins) WN DB)) AND ({english} WN LA)) AND (1990-* WN YR)) |
| **Proquest, Biological Science Collection – Date Range 1990-2023** |
| {ti(antimicrobial OR anti-microbial OR antibiotic OR anti-biotic OR antibacterial OR anti-bacterial) AND ti(resistance OR susceptibility)} AND (ti(dashboard OR application OR "web-based tool" OR "web based tool" OR interactive OR visuali?ation OR display OR decision support OR decision-support OR {information system} OR user interface design OR user-interface design) OR ab(dashboard OR application OR "web-based tool" OR "web based tool" OR report OR reports OR interactive OR visuali?ation OR display OR decision support OR decision-support OR {information system} OR user interface design OR user-interface design)) AND (ti(surveillance OR database OR updat* OR real-time OR real time) OR ab(surveillance OR database OR updat* OR real-time OR {real time})) |
| **Ovid Technologies Inc., MEDLINE** |
| \| **#** \| **Query** \| \| --- \| --- \| \| 1 \| (antimicrob* or anti-microb* or antibiot* or anti-biot* or antibacter* or anti-bacter*).kw,ti. \| \| 2 \| exp Anti-Bacterial Agents/ \| \| 3 \| 1 or 2 \| \| 4 \| (resistance or susceptibility).kw,ti. \| \| 5 \| exp Drug Resistance, Microbial/ \| \| 6 \| 3 and 4 \| \| 7 \| 5 or 6 \| \| 8 \| (dashboard or application or interactive or visualization or display).tw. \| \| 9 \| (decision support or decision-support or information system or user interface design or user-interface design or web based tool or web-based tool).tw. \| \| 10 \| Decision Support Systems, Clinical/ or Geographic Information Systems/ or User-Computer Interface/ \| \| 11 \| Data Visualization/ \| \| 12 \| 8 or 9 or 10 or 11 \| \| 13 \| (surveillance or database or updat* or real-time or real time).tw. \| \| 14 \| exp Population Surveillance/ \| \| 15 \| 13 or 14 \| \| 16 \| 7 and 12 and 15 \| \| 17 \| limit 16 to yr="1990 -Current" \| \|  \|  \| |
