# Supplementary material for: Characterizing approaches used to display antimicrobial resistance data in veterinary and human medicine: a scoping review
Source: Antimicrob Steward Healthc Epidemiol. 2025 Dec 17;5(1):e344. doi: 10.1017/ash.2025.10243 (PMC12722559; doi:10.1017/ash.2025.10243)
Supplement: Alberts et al. supplementary material [file S2732494X2510243Xsup001.zip › S1 Text.pdf]

Characterizing Approaches Used to Display Antimicrobial Resistance Data in Veterinary and Human Medicine: A Scoping Review Protocol

Authors: Leilani Rocha[1], Sheila Keay[1], Famke Alberts[1], Theresa Bernardo[1], Kurtis Sobkowich[1], Zvonimir Poljak[1]

Author Affiliations: [1]Department of Population Medicine, Ontario Veterinary College, University of Guelph, Guelph, ON, Canada.

Abstract

Background: Complex antimicrobial resistance (AMR) data is widely collected and may be communicated through real-time systems to support stakeholders, including clinicians, policymakers, and researchers. Future developers may benefit from a review of current display methods to ensure AMR data is presented in an accurate and user-friendly manner.

Objectives: This protocol describes the methods used to conduct a scoping review answering the question: "What approaches are used to display antimicrobial resistance data in real-time for surveillance and/or clinical decision-making?"

Eligibility Criteria: Eligible publications must be available in English and published after December 31st, 1989 (i.e., 1990 and beyond). Publications must be primary research articles or conference proceedings describing the methods used to display antimicrobial resistance data regarding animals and/or humans with minimal to no time lag.

Sources of Evidence: Databases of published literature from five bibliographic platforms (MEDLINE via Ovid, Web of Science (Core Collection), Biological Science Database via ProQuest, and Compendex and INSPEC via Engineering Village), selected conference proceedings, and hand-search of references from identified key review papers.

Charting Methods: Data will be characterized based on publication information, data used, display methodology, and intended users.

1. Introduction

1.1 Rationale

Antimicrobial resistance is a major health challenge for human and animal populations and is a complex One Health problem. Results from a comprehensive systemic analysis conducted at the global level, indicated that 1.27 million (95% Uncertainty Interval= 0·911, 1·71) human deaths were attributable to bacterial AMR in 2019 alone (Antimicrobial Resistance Collaborators, 2022). These estimates were based on a counterfactual scenario in which infections with resistant bacteria were compared to infections with susceptible ones. Once the counterfactual scenario used was based on no-infections as the baseline, the estimated deaths associated with AMR in 2019 were 4.95 million (95% UI=3.62,6.57 million) (Antimicrobial Resistance Collaborators, 2022). The knowledge base for antimicrobial resistance is complex (Graham et al., 2019).. Exposure of humans and animals to microorganisms occurs through many mechanisms including oral portal of entry through vehicles such as water, food or soil, or through direct contact with other animals or people. Such exposures also include the exposure to resistant bacteria and antimicrobial resistance genes. Some AMR is inherent, while other is acquired and the mechanisms of acquiring AMR could be biologically complex; yet, regardless of the mechanism, it could result in an indistinguishable AMR phenotype. Even when sophisticated molecular methods are used, the problem does not become less complex. For example, it has been argued that the abundances of antimicrobial resistance genes are frequently lower than 0.1% of microbial DNA sources, and establishing a clear association between one or more antimicrobial resistance genes to phenotypic resistance remains difficult, in commonly present mixed microbial communities (Graham et al., 2019).

Provided the negative impact of misinformation on complex topics can be significant, it is the responsibility of communicators to ensure a robust and accurate presentation of information (Swire et al., 2018). Therefore, establishing effective approaches to display complex AMR data may be advantageous for clinical decision-making and surveillance.

Public health surveillance is defined by Thacker and Birkhead (2008) as “the ongoing, systematic collection, analysis, and interpretation of health-related data essential to planning, implementation, and evaluation of public health practice.” AMR surveillance data is widely collected and may be communicated through real-time systems to support multiple stakeholders, including clinicians, policymakers, and researchers. Examples of real-time systems include interactive dashboards or applications varying in coverage, complexity, and emphases across existing systems (Stedtfeld et al., 2016). However, available information characterizing and comparing existing approaches to real-time displays of human and veterinary AMR data may be limited. Additionally, complex and evolving AMR data increases the challenge for the interpretation of display outcomes. Time is a significant barrier for medical practitioners (Straus & Haynes, 2009) and veterinarians (Keay et al., 2020) to stay up to date with primary research. This raises concerns since research synthesis under the framework of evidence-based medicine requires time and robust steps to mitigate risk of bias (O’Connor et al., 2015). Hence, when faced with urgent decision-making, human and veterinary clinicians and the public may benefit from access to user-friendly, accurate, and interpretable displays of AMR data. These premises form the focus of this review.

Safdari et al. (2018) published a scoping review of publications current to 2016 comparing computational methods and algorithms used in AMR surveillance systems to aid in human healthcare and policy decision-making. This review will expand its purpose to include veterinary AMR data and all approaches to data display systems including non-computational methods.

This scoping review could be useful to public and private agencies and healthcare facilities aiming to communicate AMR data for risk communication and/or clinical decision-making. In addition, review findings may be used as a basis for further investigation of the nature and purpose of end-user access to AMR data display systems, evaluation of the effectiveness of AMR display systems for improving decision-making, and for further development of optimal interactive displays.

1.2 Objectives

This protocol describes the rationale and methods of a formal scoping review answering the question, "What approaches are used to display antimicrobial resistance data in real-time for surveillance and/or clinical decision-making?" This scoping review will follow the methodological framework of Arksey and O'Malley (2005).

2. Methods

2.1 Protocol and Registration

This protocol will be published in the University of Guelph collection, "The Atrium" (https://atrium.lib.uoguelph.ca). The protocol was structured using the Preferred Reporting Items for Systematic Reviews and Meta-Analyses Extension for Scoping Reviews (PRISMA-ScR) guidelines (Tricco et al. 2018).

2.2 Eligibility Criteria

Eligibility criteria are outlined in Table 1. Publications selected for this scoping review must be available in English and published during or after 1990 (date of the earliest identified eligible publication in Safdari et al. (2018)). Publications will be restricted to primary research articles and conference proceedings that describe a methodology for real-time displays of antimicrobial resistance data, where real-time is defined as a time lag of no more than quarter-yearly updates. There will be no restrictions regarding the outcomes of data collected and displayed. Unpublished opinion pieces, websites, and government reports or policies without published methodology will not be included. Review articles will be excluded but tagged.

Table 1. Summary of Inclusion/Exclusion Criteria.

**Inclusion**	**Exclusion**
Full-text available in English	Full-text not available in English
Published in 1990 or later	Published before 1990
Describes methodology for the display of antimicrobial resistance data	Does not describe the methodology for the display of antimicrobial resistance data

Display of antimicrobial resistance data is in real-time or updated with a time lag of no more than quarter-yearly	Display of antimicrobial resistance data is updated at intervals exceeding quarter-yearly
Primary research articles, conference proceedings	Reviews (i.e., narrative, or systematic), government reports and policies, websites, commentaries, opinion pieces

2.3 Information Sources

The following bibliographic databases will be searched for published literature: MEDLINE via Ovid, Web of Science (Core Collection), Biological Science Database via ProQuest, and Compendex and INSPEC via Engineering Village. Though reviews and publications solely describing surveillance systems will not be included in the criteria, they will be flagged during screening and references will be hand-searched for missed and potentially relevant publications to include in level 1 screening. In a similar manner, three key journal articles: Safdari et al. (2018), Oumou Diallo et al. (2020), and Beade et al. (2022) will be hand-searched for relevant references. Lastly, the 'snow-balling' method as described in Jalali et al. (2012) will be used to search the key AMR surveillance websites: GLASS, CIPARS, IDEXX, EARS-Net, and NARMS for relevant publications.

Conference Proceedings

The conference proceedings and abstracts outlined below will be hand-searched:

- International Society for Disease Surveillance (ISDS) Conference Abstracts - https://ojphi.org/ojs/index.php/ojphi/issue/archive
- Advisory Committee on Antimicrobial Prescribing, Resistance and Healthcare-Associated Infections (APRHAI) Annual Meetings - https://www.gov.uk/government/publications/APRHAI-annual-reports
- IEEE International Symposium on Computer-based Medical Systems (CBMS) – https://www.computer.org/csdl/proceedings/1000153
- International Conference on ICTs for Healthcare (ICICTH) - https://waset.org/icts-for-healthcare-conference

2.4 Search

Search strategies will be developed with support from the University of Guelph librarian with expertise in databases and developing scoping reviews. Search strings will be formatted for each bibliographic database. Based on the research question and inclusion criteria, keywords were categorized into three main categories outlined below: the subject of antimicrobial resistance, the display of data, and data in real-time.

Table 2. Keywords Used in Each Search Category.

**Category**	**Keywords**
AMR	antimicrob* OR anti-microb* OR antibiot* OR anti-biot* OR antibacter* OR anti-bacter*) AND (resistance OR susceptibility))
Display of data:	dashboard OR application OR interactive OR visualization OR display

Computer science approaches	decision support OR decision-support OR information system OR user interface design OR user-interface design OR web based tool OR web-based tool
Data in real-time	surveillance OR database OR updat* OR real-time OR real time

Sample Search Strategy in Ovid - MEDLINE:

#	Query	Results from June 7, 2022
1	(antimicrob* or anti-microb* or antibiot* or anti-biot* or antibacter* or anti-bacter*).kw,ti.	256,070
2	exp Anti-Bacterial Agents/	787,116
3	1 or 2	871,308
4	(resistance or susceptibility).kw,ti.	300,307
5	exp Drug Resistance, Microbial/	178,781
6	3 and 4	68,183
7	5 or 6	199,600
8	(dashboard or application or interactive or visualization or display).tw.	1,256,743
9	(decision support or decision-support or information system or user interface design or user-interface design or web based tool or web-based tool).tw.	43,480
10	Decision Support Systems, Clinical/ or Geographic Information Systems/ or User-Computer Interface/	56,336
11	Data Visualization/	527
12	8 or 9 or 10 or 11	1,331,366
13	(surveillance or database or updat* or real-time or real time).tw.	1,042,670
14	exp Population Surveillance/	74,020
15	13 or 14	1,083,956
16	7 and 12 and 15	610
17	limit 16 to yr="1990 -Current"	598

2.5 Selection of Sources of Evidence

The citations from the search results across all information sources will be downloaded to Mendeley and de-duplicated. After deduplication, the citations will be uploaded to Distiller-SR® (Copyright ©2008-2022, Evidence Partners Inc.) for relevance screening and data charting. The screening process will be conducted independently by two reviewers using study-specific forms on the Distiller-SR® software. Forms will be pre-tested by both reviewers on 10 studies selected at random. Necessary adjustments will be made for clarity prior to continuing. During screening, conflicts will be resolved by consensus, and if not resolved, a third reviewer will be consulted. The first level, or primary screening will be done at the title and abstract level for each citation. After primary screening, the full-texts for all relevant citations and all labelled as “unclear” will be uploaded and be advanced to the secondary full-text relevance screening. All relevant

publications will be advanced for data charting. Reasons for exclusion during each level of screening will be recorded.

Primary Screening Questions (Title/Abstract):

1. Is the publication available in English?
   - Yes (Neutral)
   - No (Exclude)

2. Does the publication describe the display or visualization of antimicrobial resistance data in animals and/or humans?
   - Yes (Neutral)
   - No (Neutral)
     - (If 'No') Does the publication describe a surveillance system(s) of antimicrobial resistance data in animals and/or humans?
       - Yes (Exclude – Hand-search)
       - No (Exclude)
   - Unclear (Neutral)

3. Does the publication describe the antimicrobial resistance data as real-time, frequent with time gaps of up to quarter-yearly, and/or obtained through surveillance or an updated database?
   - Yes (Neutral)
   - No (Exclude)
   - Unclear (Neutral)

4. Is the publication primary research and/or a conference proceeding?
   - Yes (Include)
   - No (Exclude)
     - (If 'No') Is the publication a review?
       - Yes (Exclude – Hand-search)
       - No (Exclude)
   - Unclear (Include)

Secondary Screening (Full-Text):

1. Is the publication available in English?
   - Yes (Neutral)
   - No (Exclude)

2. Does the publication describe the methodology for the display or visualization of antimicrobial resistance data in animals and/or humans?
   - Yes (Neutral)
   - No (Neutral)
     - (If 'No') Does the publication describe a surveillance system(s) of antimicrobial resistance data in animals and/or humans?

        - Yes (Exclude – Hand-search)
        - No (Exclude)
  - Unclear (Neutral)

3. Does the publication describe the antimicrobial resistance data as real-time or frequent with time gaps of up to quarter-yearly and/or obtained through surveillance or an updated database?
   - Yes (Neutral)
   - No (Exclude)
   - Unclear (Neutral)

4. Is the publication primary research and/or a conference proceeding?
   - Yes (Include)
   - No (Exclude)
     - (If 'No') Is the publication a review?
       - Yes (Exclude – Hand-search)
       - No (Exclude)
   - Unclear (Include)

2.6 Data charting process

Data will be characterized using Distiller-SR® (Copyright © 2008-2022, Evidence Partners Inc.). The data will be characterized independently by two reviewers using forms built in Distiller-SR®. Forms will be pre-tested independently by both reviewers on 10 studies selected at random. Any necessary adjustments to the form for clarity will be made prior to continuing. Conflicts will be discussed and resolved by a consensus, and if not resolved, a third reviewer will be consulted.

2.7 Data Items

The following proposed information will be extracted.

1. Publication Information
   a. Publication year
   b. Country of the first author
   c. First author affiliation
   d. Funding information

2. Data Used
   a. Which database(s) was used to collect AMR data?
      i. Database affiliation
      ii. Level of coverage (e.g., Site-specific, Regional, National, Supra-national, International, Not provided)
   b. What are the source species present in the data?
   c. Was the data restricted to specific bacterial species?

d. Which outcome was used as a base for the data used? (e.g., Gene resistance, Qualitative (ex. phenotypes, S/I/R), Quantitative, Not stated).
    e. Was the data restricted to specific antimicrobials?
    f. Is the data presented customizable through query filters?

3. Display Methodology
    a. What is the name of the display?
        i. Management authority of display
    b. What was the stated objective of the display?
    c. What type of data display was created?
    d. Which software(s) or programming was used in the display process?
    e. How is the display updated in real-time?
    f. Is the display currently functional as described in the paper?
    g. What information was used to display the data?
        i. Geographic information
        ii. Temporal information
        iii. Additional demographic information
    h. Which specific sample types were displayed?
    i. Were there defined protocols to validate data prior to population of the display?
    j. How was epidemiological quality regarding the previous treatment of subjects and sampling of the data considered in the methodology?

4. Intended Users
    a. What was the intended audience of the data display?
    b. Who has access to the data display?

2.8 Critical appraisal

Publications included in this scoping review will not be critically appraised.

2.9 Synthesis of Results

A PRISMA flow diagram will be used to display the number of publications cleared in each stage of screening. Charted data will be grouped and summarized through frequency and summary tables, text, and figures (graphs) when applicable.

2.10 Limitations

Approaches to AMR data display are limited to those published with the inclusion of methodology. Unpublished approaches may be readily available through various platforms such as websites, however, were not identified with our search strategy. Thus, it is important to recognize that information regarding some display methods may be underrepresented in the research literature.

3. Funding

This work is supported (in part or in full) through a cooperative agreement between USDA APHIS and Cornell University's College of Veterinary Medicine (FAIN: AP23VSSP0000C114). Additional funding is provided by the Natural Sciences and Engineering Research Council of Canada (NSERC) through the Undergraduate Student Research Assistantship program.

References

Antimicrobial Resistance Collaborators. Global burden of bacterial antimicrobial resistance in 2019: a systematic analysis. Lancet. 2022 Feb 12;399(10325):629-655. doi: 10.1016/S0140-6736(21)02724-0. Epub 2022 Jan 19.

Arksey, H., & O'Malley, L. (2005). Scoping studies: towards a methodological framework. *International Journal of Social Research Methodology*, *8*(1), 19–32. https://doi.org/10.1080/1364557032000119616

Baede, V. O., David, M. Z., Andrasevic, A. T., Blanc, D. S., Borg, M., Brennan, G., Catry, B., Chabaud, A., Empel, J., Enger, H., Hallin, M., Ivanova, M., Kronenberg, A., Kuntaman, K., Larsen, A. R., Latour, K., Lindsay, J. A., Pichon, B., Santosaningsih, D., & Schouls, L. M. (2022). MRSA surveillance programmes worldwide: moving towards a harmonised international approach. *International Journal of Antimicrobial Agents, 59*, 106538. https://doi.org/10.1016/j.ijantimicag.2022.106538

Diallo, O. O., Baron, S. A., Abat, C., Colson, P., Chaudet, H., & Rolain, J.-M. (2020). Antibiotic Resistance Surveillance Systems: A Review. *Journal of Global Antimicrobial Resistance*, *23*, 430–438. https://doi.org/10.1016/j.jgar.2020.10.009

Graham, D. W., Bergeron, G., Bourassa, M. W., Dickson, J., Gomes, F., Howe, A., Kahn, L. H., Morley, P. S., Scott, H. M., Simjee, S., Singer, R. S., Smith, T. C., Storrs, C., & Wittum, T. E. (2019). Complexities in understanding antimicrobial resistance across domesticated animal, human, and Environmental Systems. *Annals of the New York Academy of Sciences*, *1441*(1), 17–30. https://doi.org/10.1111/nyas.14036

Jalali, S., & Wohlin, C. (2012). Systematic literature studies: database searches vs. backward snowballing. Proceedings of the ACM-IEEE International Symposium on Empirical Software Engineering and Measurement, 29–38. https://doi.org/10.1145/2372251.2372257

Keay, S., Sargeant, J. M., O'Connor, A., Friendship, R., O'Sullivan, T., & Poljak, Z. (2020). Veterinarian barriers to knowledge translation (kt) within the context of swine infectious disease research: An International Survey of swine veterinarians. *BMC Veterinary Research*, *16*(1). https://doi.org/10.1186/s12917-020-02617-8

O'Connor, A., & Sargeant, J. (2015). Research synthesis in veterinary science: Narrative reviews, systematic reviews and meta-analysis. *The Veterinary Journal*, *206*(3), 261–267. https://doi.org/10.1016/j.tvjl.2015.08.025

Safdari, R., GhaziSaeedi, M., Masoumi-Asl, H. Rezaei-Hachesu, P. Mirnia, K. Samad-Soltani, T. (2020). Knowledge discovery and visualization in antimicrobial resistance surveillance systems: a scoping review. *Artificial Intelligence Review*, 53, 369–406. https://doi.org/10.1007/s10462-018-9659-6

Stedtfeld, R. D., Williams, M. R., Fakher, U., Johnson, T. A., Stedtfeld, T. M., Wang, F., Khalife, W. T., Hughes, M., Etchebarne, B. E., Tiedje, J. M., & Hashsham, S. A. (2016). Antimicrobial resistance dashboard application for mapping environmental occurrence and resistant pathogens. *FEMS Microbiology Ecology*, *92*(3), fiw020. https://doi.org/10.1093/femsec/fiw020

Straus, S., & Haynes, R. B. (2009). Managing evidence-based knowledge: the need for reliable, relevant and readable resources. *CMAJ : Canadian Medical Association Journal*, *180*(9), 942–945. https://doi.org/10.1503/cmaj.081697

Swire, B., & Ecker, U. (2018). Misinformation and Its Correction: Cognitive Mechanisms and Recommendations for Mass Communication. *Misinformation and Mass Audiences*, 195–211. https://doi.org/10.7560/314555-013

Thacker, S. B., & Birkhead G.S. Surveillance. In: Gregg, M. B. (2008). *Field epidemiology*. Oxford University Press.

Tricco, A. C., Lillie, E., Zarin, W., O'Brien, K. K., Colquhoun, H., Levac, D., Moher, D., Peters, M. D. J., Horsley, T., Weeks, L., Hempel, S., Akl, E. A., Chang, C., McGowan, J., Stewart, L., Hartling, L., Aldcroft, A., Wilson, M. G., Garritty, C., & Lewin, S. (2018). PRISMA Extension for Scoping Reviews (PRISMA-ScR): Checklist and Explanation. *Annals of Internal Medicine*, *169*(7), 467. https://doi.org/10.7326/m18-0850
