# Supplementary material for: Characterizing approaches used to display antimicrobial resistance data in veterinary and human medicine: a scoping review
Source: Antimicrob Steward Healthc Epidemiol. 2025 Dec 17;5(1):e344. doi: 10.1017/ash.2025.10243 (PMC12722559; doi:10.1017/ash.2025.10243)
Supplement: Alberts et al. supplementary material [file S2732494X2510243Xsup001.zip › S11 Table.docx]

**S11 Table. Bibliographic databases and platforms (vendor interfaces) that were searched.**

| **Platform (Vendor Interface)** | **Database** |
| --- | --- |
| Clarivate | Web of Science Core Collection |
| Elsevier | Engineering Village- Inspec and Compendex |
| Ovid Technologies Inc. | MEDLINE |
| ProQuest | Biological Science Database |
