# Supplementary material for: Characterizing approaches used to display antimicrobial resistance data in veterinary and human medicine: a scoping review
Source: Antimicrob Steward Healthc Epidemiol. 2025 Dec 17;5(1):e344. doi: 10.1017/ash.2025.10243 (PMC12722559; doi:10.1017/ash.2025.10243)
Supplement: Alberts et al. supplementary material [file S2732494X2510243Xsup001.zip › S12 Table.docx]

**S12 Table. Conference proceedings that were hand-searched for relevant abstracts relating to the criteria in the scoping review.**

| **Conference** | **Proceeding Availability** |
| --- | --- |
| International Society for Disease Surveillance (ISDS) | Annual international conference, dismantled in 2019. |
| Advisory Committee on Antimicrobial Prescribing, Resistance and Healthcare-Associated Infections (APRHAI) | Annual meetings from the Department of Health and Social Care in England. Annual reports available for 2012-2017. |
| IEEE International Symposium on Computer-based Medical Systems (CBMS) | Annual international conference. Titles and full proceedings available for 1988-2024. |
| International Conference on ICTs for Healthcare (ICICTH) | Annual international conference. |
