# Supplementary material for: Characterizing approaches used to display antimicrobial resistance data in veterinary and human medicine: a scoping review
Source: Antimicrob Steward Healthc Epidemiol. 2025 Dec 17;5(1):e344. doi: 10.1017/ash.2025.10243 (PMC12722559; doi:10.1017/ash.2025.10243)
Supplement: Alberts et al. supplementary material [file S2732494X2510243Xsup001.zip › S2 Table.docx]

S2 Table: Forms used for screening and data charting.

| **Level 1 – Title/Abstract Screening** | | |
| --- | --- | --- |
| Question | Answers | Effect |
| 1. Is the abstract available in English? | Yes | Continue to Q2 |
|  | No | Exclude |
| 2. Does the publication describe the display or visualization of antimicrobial resistance data in animals and/or humans? | Yes | Continue to Q3 |
|  | No | Exclude, continue to Q2a |
|  | Unclear | Continue to Q3 |
| 2a. Does the publication describe a specific surveillance system(s) of antimicrobial data in animals and/or humans? | Yes | Exclude, flag as surveillance |
|  | No | Exclude |
|  | Unclear | Exclude, flag as surveillance |
| 3. Does the publication describe the AMR data as real-time, frequent with time gaps of up to quarter-yearly, and/or obtained through surveillance or updated database? | Yes | Continue to Q4 |
|  | No | Exclude |
|  | Unclear | Continue to Q4 |
| 4. Is the publication primary research and/or a conference proceeding? | Yes | Include in Level 2 |
|  | No | Exclude, continue to Q4a |
|  | Unclear | Include in Level 2 |
| 4a. Is the publication a review? | Yes | Exclude, flag as review |
|  | No | Exclude |

| **Level 2- Full-Text Screening** | | |
| --- | --- | --- |
| Question | Answer | Effect |
| 1. Is the full body text (beyond title/abstract) available in English? | Yes | Continue to Q2 |
|  | No | Exclude |
| 2. Does the publication describe the methodology for the display or visualization of antimicrobial resistance data in animals and/or humans? | Yes | Continue to Q3 |
|  | No | Exclude, continue to Q2a |
|  | Unclear | Continue to Q3 |
| 2a. Does the publication describe a specific surveillance system(s) of antimicrobial data in animals and/or humans? | Yes | Exclude, flag as surveillance |
|  | No | Exclude |
|  | Unclear | Exclude, flag as surveillance |
| 3. Does the publication describe the AMR data as real-time or frequent with time gaps of up to quarter-yearly? | Yes | Continue to Q4 |
|  | No | Exclude |
|  | Unclear | Continue to Q4 |
| 4. Is the publication primary research and/or a conference proceeding? | Yes | Include in Level 2 |
|  | No | Exclude, continue to Q4a |
|  | Unclear | Include in Level 2 |
| 4a. Is the publication a review? | Yes | Exclude, flag as review |
|  | No | Exclude |

| **Data Charting** | | |
| --- | --- | --- |
| Question | Answer | Effect |
| 1. Ref ID | The number provided by Distiller |  |
| **Publication Information** | | |
| 2. Year of Publication | YYYY (ex. 2021) |  |
| 3. What country is the first author affiliated with? | Select all that apply. If not stated select "Not Stated". |  |
| 4. What is the first author affiliation? | Select all that apply. If not stated select "Not Stated". If the answer was not present add it to list. |  |
| 5. What is the funding source? | Copy and paste from publication. |  |
| **Data Used** | | |
| 6. What is the name of the specific database(s) used to collect AMR data? | Select all that apply. If not stated select "Not Stated". If the answer was not present add it to list. I weblink available copy and paste weblink. |  |
| 7. What is the total geographic level of coverage of the database(s)? | Select all that apply. If not stated select "Not Stated". If the answer was not present add it to list. |  |
| **Source Species** | | |
| 8. What are the source species present in the data used? | Select all that apply. If not stated select "Not Stated". If the answer was not present add it to list. | If a non-human species is selected Q9 becomes available. |
| 9. Was the database restricted to a classification of animal species? | Select all that apply. If not stated select "Not Stated". If the answer was not present add it to list. |  |
| **Bacterial Species** | | |
| 10. Was the data restricted to specific bacterial genera and/or species? | Select all that apply: |  |
|  | Genera-specific | Q11 and Q12 become available |
|  | Species-specific | Q13 and Q14 become available |
|  | No restrictions |  |
|  | Not stated |  |
| 11. How many bacterial genera was the data restricted to? | Type number. |  |
| 12. What are the bacterial genera present in the data used? | Select all that apply. If not stated select "Not Stated". If the answer was not present add it to list. |  |
| 13. How many bacterial species are present in the data used? | Type number. |  |
| 14. What are the bacterial species present in the data used? | Select all that apply. If not stated select "Not Stated". If the answer was not present add it to list. |  |
| **Antimicrobial Resistance** | | |
| 15. Which outcome was used as a base for the data used? | Select all that apply: |  |
|  | Gener resistance |  |
|  | Qualitative |  |
|  | Quantitative |  |
|  | Not stated |  |
| 16. Was the data restricted to specific antimicrobials? | Yes | Q17 becomes available |
|  | No |  |
|  | Not stated |  |
| 17. How many antimicrobials or antimicrobial groups are present in the data used? | Type the number. |  |
| 18. Are there additional comments of note regarding the number of antimicrobials present in data? | Type any notes, if there are no notes then skip. |  |
| 19. Is the data presented in the display customizable through query filters? | Yes |  |
|  | No |  |
|  | Not stated |  |
| **Display Methodology** | | |
| 20. What is the name of the display? | Type name and if available weblink. If not stated type “Not stated”. |  |
| 21. Stated objective of the display | Copy and paste from the abstract/introduction. If not stated type “Not stated” |  |
| 22. Who maintains management authority of the display | Select all that apply. If not stated select "Not Stated". If the answer was not present add it to list. |  |
| 23. What type of data display was created according to the author(s)? | Select all that apply. If not stated select "Not Stated". If the answer was not present add it to list. |  |
| 24. Which software(s) and/or libraries were used in the display process? | Select all that apply. If not stated select "Not Stated". If the answer was not present add it to list. |  |
| 25. How is the display updated? | Automatic |  |
|  | Manual |  |
|  | Not stated |  |
| 26. Is the display currently functional as described in the paper? | Yes |  |
|  | No |  |
|  | Functional, but exceeds quarter-yearly | Q27 becomes available. |
|  | Unknown (not findable) |  |
| 27. In what year was the display last updated? | Type number, if unknown type Not stated. |  |
| 28. How was the data displayed geographically? | Select all the apply: |  |
|  | Not stated |  |
|  | Country |  |
|  | State/province |  |
|  | Region |  |
|  | Exact location (GPS) |  |
|  | Primary care organization |  |
|  | Other |  |
| 29. How was the data displayed temporally? | Select all that apply. If not stated select "Not Stated". If the answer was not present add it to list. |  |
| 30. Were specific sample type(s) displayed? | Yes | Q31 becomes available. |
|  | No |  |
|  | Not stated |  |
| 31. Was the display restricted to specific sample types | Yes | Q32 and Q33 become available. |
|  | No |  |
|  | Not stated |  |
| 32. How many specific sample types were displayed with the data? | Type a number, or type “Not stated”. |  |
| 33. Which specific sample types were displayed with the data? | Select all that apply. If not stated select "Not Stated". If the answer was not present add it to list. |  |
| 34. How was epidemiological quality regarding previous treatment of subjects and sampling of the data considered in the methodology? | Select all that apply. If not stated select "Not Stated". If the answer was not present add it to list. |  |
| **Intended Users:** | | |
| 35. What was the stated intended audience of the data display? | Select all that apply. If not stated select "Not Stated". If the answer was not present add it to list. |  |
| 36.Who has access to the data display? | Not Stated |  |
|  | Public |  |
|  | Authorized users |  |
| 37. Are there additional comments of note regarding end user access for the display? | Type additional comments or skip if no additional comments |  |
