# Supplementary material for: Characterizing approaches used to display antimicrobial resistance data in veterinary and human medicine: a scoping review
Source: Antimicrob Steward Healthc Epidemiol. 2025 Dec 17;5(1):e344. doi: 10.1017/ash.2025.10243 (PMC12722559; doi:10.1017/ash.2025.10243)
Supplement: Alberts et al. supplementary material [file S2732494X2510243Xsup001.zip › S3 Table.docx]

**Table S3** Display name, objective, and funding for each included publication. Per PRISMA guidelines, a summary of each included publication has been provided. The objective is as reported in the publication abstract or introduction. The display name and access point are as reported in the publication. The funding reported for each publication is included in the scoping review as required per PRISMA guidelines. If no display name, objective, or funding was listed then it is “Not Stated.”

| **Reference** | **Display Name** | **Objective** | **Funding** |
| --- | --- | --- | --- |
|  |  |  |  |
| Altorf-van der Kuil et al., 2017 | ISISWeb, https://www.isis-web.nl/ | The main objectives are to monitor the magnitude and trends of AMR as well as outbreaks involving AMR to retrospectively identify or prospectively monitor the emergence of new AMR mechanisms based on their phenotypic expression. Data supports policy making in public health and healthcare, the development of antibiotic treatment guidelines, and to facilitate research, to contribute to quality, safety and reduction of healthcare costs. | The national AMR surveillance system is supported by the Dutch Ministry of Health. |
| Argimón et al., 2021 | Typhi Pathogenwatch, https://pathogen.watch/styphi | Typhi Pathogenwatch, a web application to support genomic epidemiology and public health surveillance of S. Typhi. Typhi Pathogenwatch rapidly places new genomes within the broader geographic and population context, predicts their genotype according to established nomenclatures and detects the presence of AMR determinants and plasmid replicon genes to assess public health risk. | Pathogenwatch is developed with support from Li Ka Shing Foundation (Big Data Institute, University of Oxford) and Wellcome (grant number 099202). S.A. and D.M.A. are supported by the National Institute for Health Research (UK) Global Health Research Unit on genomic Surveillance of AMR (16_136_111) and by the Centre for Genomic Pathogen Surveillance (http:// pathogensurveillance.net). Z.A.D. received funding from the European Union’s Horizon 2020 research and innovation programme under the Marie SkłodowskaCurie grant agreement TyphiNET No 845681. L.S.B. is funded by Plan GenT (CDEI06/20-B), Conselleria de Sanitat Universal i Salut Pública, Generalitat Valenciana (Valencia, Spain). |
| Barquero et al., 2022 | KARGAMobile, https://github.com/Ruiz-HCI-Lab/ KargaMobile | In this paper, we present KARGAMobile, a mobile app for portable, real-time, easily interpretable analysis of ARGs from Nanopore sequencing data. | This work is in part supported by US grants: NIH NIAID R01AI145552; NSF SCH 2013998. |
| Bohanec et al., 1997 | Podpora terapevrskih aktivnosti pri hospitalnih infekcijah (Ptah) | The system is designed as an on-line tool for a medical doctor. It performs four types of analyses of micro-biological findings regarding bacterial resistance to antibiotics and effectiveness of antibiotics. | The work reported here was financially supported by the Ministry of Science and Technology of the Republic of Slovenia. |
| Boussat et al., 2012 | ConsoRes, https://www.consores.net/ | Information on the relationship between antibiotic consumption and bacterial resistance is not fully documented. A collection system should thus be implemented locally “the closest possible to the prescriber” insuring transmission of antibiotic consumption and bacterial resistance for the various personnel of healthcare institutions so as to allow for a fine and quick analysis. The Eastern CClin developed an online tool to this end, called ConsoRes, which has been tested since January 2011 in some volunteer institutions of the greater North-East of France. | Not Stated. |
| Brossette & Hymel, 2008 | Cardinal Health Data Mining Surveillance System (DMSS) | In this article, we examine data mining in laboratory medicine and infection control and describe future opportunities in the space.Data mining, infection control,and laboratory medicine intersect at the use of clinical laboratory data by computers to automatically construct models that describe or predict hospital epidemiology patterns of statistical and clinical significance.It is difficult, however, for infection control to identify new risk threats, intervene, and track outcomes continuously, hospital-wide. These challenges can be mitigated by a properly designed data mining system. | Not Stated. |
| Criscuolo et al., 2021 | resistancebank.org, https://resistancebank.org/ | An online platform that centralizes information on AMR in animals from 1,285 surveys from LMICs that were conducted between 2000 and 2019 and include 22,403 resistance rates for pathogens isolated from chickens, cattle, sheep, and pigs. The platform provides access to individual surveys, country-level reports, and maps of AMR , is accessed via any internet browser, and enables users to upload surveys to strengthen a global database as a focal point for sharing AMR data in LMICs and to help international funders prioritize their actions. | This work was supported by the Branco Weiss Fellowship, and the Swiss National Science Foundation (SNF). |
| Deckert et al., 2015 | Canadian Integrated Program for Antimicrobial Resistance Surveillance (CIPARS) | The objective of the Canadian Integrated Program for Antimicrobial Resistance Surveillance (CIPARS) is to provide a unified approach to monitor national trends in antimicrobial resistance (AMR) and antimicrobial use (AMU) in humans and animals and to facilitate the assessment of the public health impact of antimicrobial use. | Not Stated. |
| Ekwanzala & Momba, 2022 | GenoTrack, http://www.genotrack.co.za/ | Consequently, a web application was developed to fill the gap of the environment-clinic nexus of ARGs. This study reports on the development of GenoTrack that geo-spatially map human bacterial genomes isolated from the environmental and clinical settings focusing on their sequence types and antibiotic resistance genes content. | The development of GenoTrack has been supported by the Department of Science and Technology/ the National Research Foundation through the South African Research Chairs Initiative in Water Quality and Wastewater Management (grant number UID87310) and the Tshwane University of Technology. MDE was supported by the National Research Foundation [grant numbers UID112851 and UID121222]. |
| Gómez-Palomo et al., 2016 | The MicrobDynamic system | In this study, we describe an electronic tool to provide updated knowledge on the previous bacterial causes of UTI in different clinical care settings and on their antibiotic susceptibility patterns, facilitating prediction of the usefulness of empiric treatments in each setting. | Not Stated. |
| Hendling et al., 2021 | ResiDB, https://residb.ait.ac.at/ | Here, we introduce resiDB, a web-based sequence database manager for bacteria, fungi, viruses, protozoa, invertebrate, plants, archaea, environmental and whole genome shotgun sequences. It is a tool that enables the streamlined creation of user-defined DNA sequence databases that can be used e.g. for the design of diagnostic assays. | This work was supported by the European Union’s Horizon 2020 research and innovation program [634137]. Funding for open access charge: H2020 [634137]. |
| Hulth et al., 2019 | OPEN Stewardship, www.openasp.org | To develop, evaluate, and implement a universal online platform - termed OPEN Stewardship- to promote responsible antimicrobial prescribing (antimicrobial stewardship). | We would like to thank funding sources including: the Joint Programming Initiative in Antimicrobial Resistance, The Canadian Institutes for Health Research, The Swedish Research Council, and the Israel Ministry of Health. |
| Idomir et al., 2010 | Not Stated. | The aim of the study was to analyze the results of the implementation of a WEB-based program for the surveillance of the resistance to antibiotics in the Clinical County Emergency Hospital of Braşov, Romania, and the optimization of this application. | Not Stated. |
| Ironmonger et al., 2013 | AmWeb | We have developed AmWeb, a novel web-enabled reporting tool, to allow laboratories in the region to analyse and review their own data, which are electronically submitted to the regional server. This produces both an incentive for their continued participation and a means for the local, timely monitoring of changes in AMR and the intervention of changing prescribing practice. | This work was supported by the HPA. |
| Jia et al., 2017 | Comprehensive Antibiotic Research Database (CARD), http://arpcard.mcmaster.ca | To provide a unifying resource for the antibiotic resistance community. The CARD seeks to include data describing antibiotics and their targets along with antibiotic resistance genes, associated proteins, and antibiotic resistance literature. | Canadian Institutes of Health Research; Natural Sciences and Engineering Research Council; Canada Research Chair (to G.D.W.’s laboratory); A.G.M. holds a Cisco Research Chair in Bioinformatics, supported by Cisco Systems Canada, Inc., and his AMR research is funded by the Canadian Foundation for Innovation, Genome Canada, Canadian Institutes of Health Research; Natural Sciences and Engineering Research Council (Canada); McMaster University Faculty of Science Interdisciplinary Research Fund; M.G. DeGroote Institute for Infectious Disease Research Summer Student Fellowship (to B.A.L.); Genome Canada/Genome BC (to F.S.L.B., R.L., M.C.); McMaster Service Lab and Repository (MSLR) computing cluster, funded in part by a grant from the Canadian Foundation for Innovation [34531 to A.G.M.]; USDA Project Numbers 6040-32000-006-00 and 6040-32000-009-00 (to J.G.F.); Postdoctoral Research Associate Fellowship awarded by the USDA, Agricultural Research Service Headquarters (to L.E.W.). Funding for open access charge: internal funds. |
| Johnson et al., 2016 | Fingertips, https://fingertips.phe.org.uk/profile/amr-local-indicators | The aim of the AMR local indicators profile on Fingertips is to support the development of local action plans to optimize antibiotic prescribing and reduce AMR and healthcare-associated infections. Provision of access to relevant information in an easy to use format will help local stakeholders,including healthcare staff, commissioners, Directors of Public Health, academics and the public, to benchmark relevant local AMR data and to monitor the impact of local initiatives to tackle AMR over time. | All work was performed as part of the routine work of the contributing authors. |
| Kaur et al., 2021 | ICMR’s Antimicrobial Resistance Surveillance system (i-AMRSS) | In this paper we present an overview of Indian Council of Medical Research (ICMR)’s Antimicrobial Resistance Surveillance system (i-AMRSS), a promising tool for collection, management and analysis of the AMR data. | This study was funded by Indian Council of Medical Research, New Delhi [ICMR File No BIC/14(01)/TF/2014]. |
| Li et al., 2020 | Described only as 'China's AMR monitoring system'. | Proposed system to provide AMR visual query and analysis functions based on derived drug resistance index and a disease assessment model using data for AMR risk assessment and monitoring. | This research was funded by the National Key Research and Development Project of China, grant number 2016YFD0501304. |
| Lim et al., 2020 | AutoMated tool for Antimicrobial resistance Surveillance System (AMASS), https://www.amass.website/ | To support clinical microbiology laboratories to analyze their microbiology and hospital data files (in CSV or Excel format) onsite and promptly generate AMR surveillance reports (in PDF and CSV formats). | Mahidol Oxford Tropical Medicine Research Unit (MORU) is funded by the Wellcome Trust (grant: 106698/Z/14/Z). Oxford University Clinical Research Unit (OUCRU) is funded by the Wellcome Trust (grant: 106680/B/14/Z). The investigators are funded by the Wellcome Trust (CLim is funded by a Training Research Fellowship [grant: 206736] and DL is funded by an Intermediate Training Fellowship [grant: 101103]). BC is funded by the UK Medical Research Council and Department for International Development (grant: MR/K006924/1). The funders of the investigators and study had no role in the study, data collection, data analysis, data interpretation, or writing of the manuscript. |
| Liu & Pop, 2009 | Antibiotic Resistance Genes Database (ARDB), http://ardb.cbcb.umd.edu/ | To address the limitations of currently available public resources, and to facilitate the identification and characterization of antibiotic resistance genes, we have created a manually curated database [Antibiotic Resistance Genes Database (ARDB)] unifying most of the publicly available genes and related information, (i) to provide a centralized compendium of information on antibiotic resistance; (ii) to facilitate the consistent annotation of resistance information in newly sequenced organisms; and (iii) to facilitate the identification and characterization of new genes. | Uniformed Services University of the Health Sciences, administered by the Henry Jackson Foundation (HU001-06-1-0015 to M.P.). Funding for open access charge: Uniformed Services University of the Health Sciences, administered by the Henry Jackson Foundation (HU001-06-1-0015). |
| MacFadden et al., 2016 | ResistanceOpen, www.resistanceopen.org | To fill gaps in traditional antimicrobial surveillance systems, we developed a platform that captures the diverse and fragmented antimicrobial resistance data that are being generated daily and are made available via the Internet. Our ultimate aim is to use this tool to better understand the global burden and geographic distribution of antimicrobial resistance. | Not Stated. |
| Majlander et al., 2021 | ResistApp, https://app.resistomap.com/sampling/332 | This study investigated the prevalence and abundance of ARGs in hospital wastewater over time using a novel digital platform, ResistApp (Resistomap Oy, Helsinki, Finland). Resist- App combines culture-independent, high-throughput gene quantification with the efficiency of automated data analysis to synthesize and visualize monitoring data in an interactive dashboard.ResistApp allows users to view detailed information on the prevalence and abundance of ARGs and genes associated with mobile genetic elements (MGEs), integrons, and bacteria causing hospital-acquired infections (HAIs) in hospital wastewater focusing on carbapenem resistance genes. | The project was supported by Business Finland R&D Funding (Project No. 287/31/2020). The funder of the study played no role in study design, data collection, data analysis, data interpretation, writing of the report, or submission of the report for publication. |
| Pinto et al., 2016 | HAITool, http://haitool.ihmt.unl.pt/ | The main goal was to design and implement an information system - HAITooL - which could effectively be a tool for the HAI and AR surveillance, being as well a decisionsupport system for antibiotics prescription. It aims at impacting on antimicrobial resistant HAI and antibiotic use, by leveraging the HP’s work. | This work was supported by project “HAITooL - A Toolkit to Prevent, Manage and Control Healthcare-Associated Infections in Portugal” EEA Grants, 000182DT3; and by FCT for funds to GHTM – UID/Multi/04413/2013. |
| Pittet et al., 1996 | Not Stated. | To describe the role of an expert system applied to the control of MRSA at a large medical center (1,600 beds) with high endemic rates.Among available patient databases, clinical microbiology laboratory and admission-discharge-transfer (ADT) databases are used to generate computer alerts. A laboratory alert (lab alert) is printed daily in the Infection Control Program (ICP) offices, listing all patients with cultures positive for MRSA detected within the preceding 24 hours. | Not Stated. |
| Rezaei-hachesu et al., 2018 | Iranian Antimicrobial Resistance (AMR) Surveillance System | To design an AMR surveillance system for use in the NICUs of northwestern Iranian hospitals. This system considers the core elements of biosurveillance systems, including data collection, analysis (statistical and intelligent), and reporting (using dashboards and geographical information systems [GIS]) modules. The developed system is fully compatible with GLASS. | Not Stated. |
| Rodriguez-Maresca et al., 2014 | Guia Electronica de Resistencias Bacterianas (GERB) | The objectives of our study were to design, develop, and implement a new computer application based on the local epidemiologic analysis of bacterial susceptibility to antibiotics and to assess the usefulness to physicians of the information that it offers for selecting the most appropriate antibiotic treatment in ICU patients with suspicion of nosocomial infection. | This study was developed within the Research Project “Analisis de los niveles de antibi ´ oticos y su aplicaci ´ on en ´ las gu´ıas electronicas de resistencias como estrategia para ´ optimizar su uso cl´ınico” (P108/90354) funded by the Carlos 8 BioMed Research International III Health Institute of the Spanish Ministry of Health through the Fondo de Investigacion Sanitaria. |
| Rolfhamre et al., 2004 | SmiNet, http://www.smittskyddsinsti | In this article we describe the considerations and technology behind a newly introduced public web tool in Sweden for easy retrieval of county and national surveillance data on communicable diseases, given as tables, maps, and trend curves. | Not Stated. |
| Saha et al., 2015 | u-CARE, http://www.ebioinformatics.net/ucare | To cater to the needs of researchers working in the field of antimicrobial drug resistance with minimal knowledge of bioinformatics. This database is also intended as a guide book to medical practitioners to avoid use of antibiotics against which resistance has already been reported in E. coli. | Not Stated. |
| Sandmann et al., 2023 | GEFAAR, https://gefaar.uni-muenster.de/ | Easy access to antimicrobial resistance data and meaningful visualization is essential to guide the empirical antimicrobial treatment and to promote the rational use of antimicrobial agents. Currently available solutions are commonly externally hosted, centralized systems. However, there is a need for close monitoring by local analysis tools. To fill this gap, we developed GEFAAR—a generic framework for the analysis of antimicrobial resistance data. Following the example of the German Robert Koch Institute (RKI), an interactive web-application is provided to determine basic pathogen and resistance statistics. In addition to the RKI’s externally maintained database, our application provides a generic framework to import tabular data and to analyze them safely in a local environment. Moreover, our application offers an intuitive web-based user interface to visualize resistance trend analysis as well as advanced cluster analyses on species- or clinic/unit level to generate alerts of potential transmission events. | Open Access funding enabled and organized by Projekt DEAL. |
| Schindler et al., 1998 | World Antibiotic Resistance Network (WARN), http ://www.warn.cs.czI | The aim of the project is to set up an information system accessible via the Internet to answer the general need of dissemination of important information world-wide and to keep microbiologists, epidemiologists, and infectionists informed about current problems of the resistance of bacteria to antibiotics. The main goal is to bring information about the current state of resistance in particular regions of the world to regions and countries distant from the world centres of research and opinion makers. | The project is supported by a grant of the Government Agency of the Czech Republic GA CR 310/96/0588. |
| Stedtfeld et al., 2016 | The AR Dashboard App | For geospatial mapping of ARGs, mobile genetic elements (MGEs) and ARB occurrence in environmental and clinical samples. | The research was funded in part by the Pharmaceuticals in the Environment Initiative, the Center for the Health Impacts of Agriculture (CHIA), Michigan State University Clinical Translational Science Institute Seed Grant App ID 224, and the Environmental Protection Agency Great lakes Restoration Initiative (GL-00E01127-0), the Superfund Research Program (2 P42 ES004911-22A1) from the National Institute for Environmental Health Sciences. |
| Stelling & Brien, 2016 | WHONET, www.whonet.org | WHONET has had two major objectives. The first is to analyze the reports of microbiology laboratories to delineate the specific problems with infections seen in health centers and the communities they serve. The aim is to provide infection control, antimicrobial stewardship committees, and public health professionals with the data they need to develop appropriate interventions to stem the spread of antimicrobial resistance. WHONET’s second objective is to enable multiple laboratories to merge their reports into multicenter, national, or global files to track the spread of antimicrobial-resistant organisms and support collaborative efforts to manage and contain them. With modern advances in Web-based information technology, WHONET supports the WHO Global Action Plan for Containment of Antimicrobial Resistance to build a global collaborative for antimicrobial resistance surveillance. | Not Stated. |
| Steurbaut et al., 2010 | Computer-based Surveillance and Alerting of Nosocomial Infections, Antimicrobial Resistance and Antibiotic Consumption (COSARA) | The aim of the present study was to enable Computer-based Surveillance and Alerting of Nosocomial Infections, Antimicrobial Resistance and Antibiotic Consumption (COSARA) in the ICU by providing computerized support at the point of care. The COSARA platform will be used in the daily follow up of infections and antibiotic therapies. It has the potential to improve the quality of care, time efficiency and cost-effectiveness of critical care infection control. | The work was supported by the Institute for the Promotion of Innovation through Science and Technology in Flanders. |
| Teodoro et al., 2012 | Antimicrobial Resistance Trend Monitoring System (ARTEMIS), http://babar.unige.ch:8080/artemis | To design and implement an architecture that can provide real-time and source-independent antimicrobial resistance monitoring to support transnational resistance surveillance. In particular, we investigated the use of a Semantic Web-based model to foster integration and interoperability of interinstitutional and cross-border microbiology laboratory databases. To fulfill these aims, we designed the Antimicrobial Resistance Trend Monitoring System (ARTEMIS). | This work is funded by the DebugIT project of the European Union Seventh Framework Programme grant agreement ICT-2007.5.2-217139. |
| K. A. Thursky et al., 2006 | Antibiotic Decision support for the Victorian Infectious Diseases Service (ADVISE) | To implement and evaluate the effect of a computerized decision support tool on antibiotic use in an intensive care unit (ICU).pg. 225. We describe the evaluation of an ICU-based decision support system that provides real-time microbiology results and patient-specific antibiotic recommendations for clinical isolates. We hypothesized that the Antibiotic Decision support for the Victorian Infectious Diseases Service (ADVISE) would significantly influence the pattern of ICU antibiotic use. | This work was funded by the Quality Branch, Department of Human Services, Victoria, Australia. |
| K. Thursky et al., 2009 | Guidance DS | Our mission was to create a generic web-based tool that allowed clinicians and pharmacists to create, update and manage electronic guidelines and restricted drug approvals for their own institutions. The aim was to support good clinical governance and improve prescribing. Guidance DS would be developed as a long-term sustainable model for guideline based CDSS that can be interfaced with existing clinical information systems. | Not Stated. |
| Tsutsui & Suzuki, 2018 | Japan Nosocomial Infections Surveillance (JANIS), https://janis.mhlw.go.jp/english/index.asp# | Aimed to propose a model of sustainable national AMR voluntary surveillance which overcame barriers of dispersed hospitals with independent microbiology laboratories and creates national reports and benchmarking reports for each hospital to facilitate infection control practices. | Research Program on Emerging and Re-emerging Infectious Diseases from the Japan Agency for Medical Research and Development (grant numbers 17fk0108307j0103) and JSPS KAKENHI Grant Number JP 15 K08863. |
| Vatopoulos et al., 1999 | WHONET - Greece, http://www.mednet.gr/whonet | To satisfy the urgent need for an efficient surveillance system to monitor the possible impact of this policy, and to study the epidemiology of antimicrobial resistance, we launched a project in 1995 to establish a national network for continuous monitoring of such resistance. | The Network described in the article has been supported,in part,by a grant from the Greek Ministry of Health and Welfare. |
| Wallace & Damani, 2013 | Not Stated. | The aim of our project was to develop a system to ensure timely communication of surveillance information to both clinical and non-clinical teams using an electronic dashboard. | Not Stated. |
| Wang et al., 2022 | VRprofile2, https://tool2-mml.sjtu.edu.cn/Vrprofile | Here, we report the release of VRprofile version 2.0, which offers three major enhancements: (i) graphical representation of the multiple resistance regions with mosaic structures and comparison to the known MGEs with similar architecture; (ii) aid to explore the relationships of antibiotic resistance genes, mobile elements, and host strains; (iii) pre-computed mobilome of >5500 ESKAPEE bacterial genomes. We expect that VRprofile2 will provide better support for researchers interested in bacterial diverse mobile elements and the dissemination of antibiotic resistance. | National Key Research and Development Program of China [2018YFE0102400]; National Natural Science Foundation of China [32070572]; Science and Technology Commission of Shanghai Municipality [19JC1413000 and 19430750600]; Medicine and Engineering Interdisciplinary Research Fund of Shanghai Jiao Tong University [19x190020171]. Funding for open access charge: National Key Research and Development Program of China [2018YFE0102400]. |
| Yadav et al., 2023 | District health information system 2 (DHIS2), https://np.amr.health/ | The objective of this project was to develop a platform for One Health AMR surveillance that would enable the rapid data aggregation, visualization, and analysis of both human and animal health data. Herein, we describe our experiences in the development of reliable, convenient, efficient, and innovative digital tools for One Health AMR surveillance data management in Nepal that can be used to generate high-quality outputs and better inform policymaking to combat AMR. | NPHL and CVL received no external funding for this project. |
| Yin et al., 2023 | ARGSs-OAP v3.0 | Continuous improvement of this analytic tool, the SARG-based ARGs-OAP, is required to advance its performance and integration with other downstream analyses. Thus, this study describes recent updates to the ARGs-OAP v3.0. | The work was substantially supported by a Theme-based Research Scheme grant from the Research Grants Council of the Hong Kong Special Administrative Region, China (T21-705/20-N) |

References

Altorf-van der Kuil, W., Schoffelen, A. F., de Greeff, S. C., Thijsen, S. F., Alblas, H. J., Notermans, D. W., Vlek, A. L., van der Sande, M. A., & Leenstra, T. (2017). National laboratory-based surveillance system for antimicrobial resistance: a successful tool to support the control of antimicrobial resistance in the Netherlands. *Eurosurveillance*, *22*(46), 17–26. https://doi.org/10.2807/1560-7917.ES.2017.22.46.17-00062

Argimón, S., Yeats, C. A., Goater, R. J., Abudahab, K., Taylor, B., Underwood, A., Sánchez-Busó, L., Wong, V. K., Dyson, Z. A., Nair, S., Park, S. E., Marks, F., Page, A. J., Keane, J. A., Baker, S., Holt, K. E., Dougan, G., & Aanensen, D. M. (2021). A global resource for genomic predictions of antimicrobial resistance and surveillance of Salmonella Typhi at pathogenwatch. *Nature Communications*, *12*(1), 2879. https://doi.org/10.1038/s41467-021-23091-2

Arya, B. K., Robert, D., Das Bhattacharya, S., & Mukhopadhyay, J. (2013). A framework for web based geographical information systems for country wide antimicrobial resistance monitoring. *Health Policy and Technology*, *2*(2), 85–93. https://doi.org/10.1016/j.hlpt.2013.03.005

Barquero, A., Marini, S., Boucher, C., Ruiz, J., & Prosperi, M. (2022). KARGAMobile: Android app for portable, real-time, easily interpretable analysis of antibiotic resistance genes via nanopore sequencing. *Frontiers in Bioengineering and Biotechnology*, *10*, 1016408. https://doi.org/10.3389/fbioe.2022.1016408

Bohanec, M., Rems, M., Slavec, S., & Urh, B. (1997). PTAH: A System for Supporting Nosocomial Infection Therapy. In *Intelligent Data Analysis in Medicine and Pharmacology* (pp. 99–111). Springer US. https://doi.org/10.1007/978-1-4615-6059-3_6

Boussat, S., Demoré, B., Lozniewski, A., Aissa, N., & Rabaud, C. (2012). How to improve the collection and analysis of hospital antibiotic consumption: Preliminary results of the ConsoRes software experimental implementation. *Médecine et Maladies Infectieuses*. https://doi.org/10.1016/j.medmal.2012.02.006

Brossette, S. E., & Hymel, P. A. (2008). Data Mining and Infection Control. *Clinics in Laboratory Medicine*, *28*(1), 119–126. https://doi.org/10.1016/j.cll.2007.10.007

Criscuolo, N. G., Pires, J., Zhao, C., & Van Boeckel, T. P. (2021). resistancebank.org, an open-access repository for surveys of antimicrobial resistance in animals. *Scientific Data*, *8*(1), 189. https://doi.org/10.1038/s41597-021-00978-9

Deckert, A., Agunos, A., Avery, B., Carson, C., Daignault, D., Finley, R., Gow, S., Léger, D., Mulvey, M., Parmley, J., Reid-Smith, R., & Irwin, R. (2015). CIPARS: A One-Health Approach to Antimicrobial Resistance Surveillance. *Online Journal of Public Health Informatics*, *7*(1). https://doi.org/10.5210/ojphi.v7i1.5734

Ekwanzala, M. D., & Momba, M. N. B. (2022). GenoTrack, a Webtool to Geospatially Link Bacterial Genomes from Environmental and Clinical Settings. *2022 IST-Africa Conference (IST-Africa)*, 1–10. https://doi.org/10.23919/IST-Africa56635.2022.9845587

Gómez-Palomo, F., Sorlózano-Puerto, A., Miranda-Casas, C., Rodríguez-Rodríguez, J. M., Navarro-Marí, J. M., & Gutiérrez-Fernández, J. (2016). Development of a web application for recording bacterial etiologic agents and their antimicrobial susceptibility to improve the treatment of urinary tract infections and monitor resistance to antibiotics. *Revista Espanola de Quimioterapia : Publicacion Oficial de La Sociedad Espanola de Quimioterapia*, *29*(2), 99–104. http://www.ncbi.nlm.nih.gov/pubmed/26964516

Hendling, M., Conzemius, R., & Barišić, I. (2021). ResiDB: An automated database manager for sequence data. *Computational and Structural Biotechnology Journal*, *19*, 847–851. https://doi.org/10.1016/j.csbj.2021.01.024

Hulth, A., Lofmark, S., Andre, J., Chorney, R., Cohn, E., Ellen, M., Davidovitch, N., Moran-Gilad, J., Greer, A., Fishman, D., Brownstein, J., & MacFadden, D. (2019). A Tool for Promoting Responsible Antibiotic Prescribing across Settings and Sectors. *Online Journal of Public Health Informatics*, *11*(1). https://doi.org/10.5210/ojphi.v11i1.9781

Idomir, M. E., Cocuz, M. E., Chesca, A., Nemet, C. G., Chesc, A., & Nemet, C. G. (2010). *Analysis of results and optimization of a WEB-based program used for the antibiotic resistance surveillance in a Romanian universitary hospital*. 215–220.

Ironmonger, D., Edeghere, O., Gossain, S., Bains, A., & Hawkey, P. M. (2013). AmWeb: a novel interactive web tool for antimicrobial resistance surveillance, applicable to both community and hospital patients. *Journal of Antimicrobial Chemotherapy*, *68*(10), 2406–2413. https://doi.org/10.1093/jac/dkt181

Jia, B., Raphenya, A. R., Alcock, B., Waglechner, N., Guo, P., Tsang, K. K., Lago, B. A., Dave, B. M., Pereira, S., Sharma, A. N., Doshi, S., Courtot, M., Lo, R., Williams, L. E., Frye, J. G., Elsayegh, T., Sardar, D., Westman, E. L., Pawlowski, A. C., … McArthur, A. G. (2017). CARD 2017: expansion and model-centric curation of the comprehensive antibiotic resistance database. *Nucleic Acids Research*, *45*(D1), D566–D573. https://doi.org/10.1093/nar/gkw1004

Johnson, A. P., Muller-Pebody, B., Budd, E., Ashiru-Oredope, D., Ladenheim, D., Hain, D., Hope, R., Bhattacharya, A., Elgohari, S., Guy, R., Henderson, K., Puleston, R., Rooney, G., Thelwall, S., Wellington, E., Lamagni, T., & Hopkins, S. (2016). Improving feedback of surveillance data on antimicrobial consumption, resistance and stewardship in England: putting the data at your Fingertips. *Journal of Antimicrobial Chemotherapy*, *72*(4), dkw536. https://doi.org/10.1093/jac/dkw536

Kaur, J., Dhama, A. S., Buttolia, H., Kaur, J., Walia, K., Ohri, V., Kumar, V., Lynn, A. M., Srivastava, A., & Singh, H. (2021). ICMR’s Antimicrobial Resistance Surveillance system ( i-AMRSS): a promising tool for global antimicrobial resistance surveillance. *JAC-Antimicrobial Resistance*, *3*(1), dlab023–dlab023. https://doi.org/10.1093/jacamr/dlab023

Li, X., Liang, B., Xu, D., Wu, C., Li, J., & Zheng, Y. (2020). Antimicrobial Resistance Risk Assessment Models and Database System for Animal-Derived Pathogens. *Antibiotics*, *9*(11), 829. https://doi.org/10.3390/antibiotics9110829

Lim, C., Miliya, T., Chansamouth, V., Aung, M. T., Karkey, A., Teparrukkul, P., Rahul, B., Lan, N. P. H., Stelling, J., Turner, P., Ashley, E., van Doorn, H. R., Lin, H. N., Ling, C., Hinjoy, S., Iamsirithaworn, S., Dunachie, S., Wangrangsimakul, T., Hantrakun, V., … Limmathurotsakul, D. (2020). Automating the Generation of Antimicrobial Resistance Surveillance Reports: Proof-of-Concept Study Involving Seven Hospitals in Seven Countries. *Journal of Medical Internet Research*, *22*(10), e19762. https://doi.org/10.2196/19762

Liu, B., & Pop, M. (2009). ARDB--Antibiotic Resistance Genes Database. *Nucleic Acids Research*, *37*(Database), D443–D447. https://doi.org/10.1093/nar/gkn656

MacFadden, D. R., Fisman, D., Andre, J., Ara, Y., Majumder, M. S., Bogoch, I. I., Daneman, N., Wang, A., Vavitsas, M., Castellani, L., & Brownstein, J. S. (2016). A Platform for Monitoring Regional Antimicrobial Resistance, Using Online Data Sources: ResistanceOpen. *The Journal of Infectious Diseases*, *214*(suppl_4), S393–S398. https://doi.org/10.1093/infdis/jiw343

Majlander, J., Anttila, V.-J., Nurmi, W., Seppälä, A., Tiedje, J., & Muziasari, W. (2021). Routine wastewater-based monitoring of antibiotic resistance in two Finnish hospitals: focus on carbapenem resistance genes and genes associated with bacteria causing hospital-acquired infections. *Journal of Hospital Infection*, *117*, 157–164. https://doi.org/10.1016/j.jhin.2021.09.008

Pinto, P., Gii, M., Simoes, A. S., Da Silva, M. M., & Lapao, L. V. (2016). HAITool - Hospital Infections Management and Decision-Making Information System. *13th European, Mediterranean and Middle Eastern Conference on Information Systems, EMCIS 2016, June 23, 2016  -  June 24, 2016*, 245–254.

Pittet, D., Safran, E., Harbarth, S., Borst, F., Copin, P., Rohner, P., Scherrer, J.-R., & Auckenthaler, R. (1996). Automatic Alerts for Methicillin-Resistant Staphylococcus aureus Surveillance and Control: Role of a Hospital Information System. *Infection Control & Hospital Epidemiology*, *17*(8), 496–502. https://doi.org/10.1086/647350

Rezaei-hachesu, P., Samad-Soltani, T., Yaghoubi, S., GhaziSaeedi, M., Mirnia, K., Masoumi-Asl, H., & Safdari, R. (2018). The design and evaluation of an antimicrobial resistance surveillance system for neonatal intensive care units in Iran. *International Journal of Medical Informatics*, *115*, 24–34. https://doi.org/10.1016/j.ijmedinf.2018.04.007

Rodriguez-Maresca, M., Sorlozano, A., Grau, M., Rodriguez-Castaño, R., Ruiz-Valverde, A., & Gutierrez-Fernandez, J. (2014). Implementation of a Computerized Decision Support System to Improve the Appropriateness of Antibiotic Therapy Using Local Microbiologic Data. *BioMed Research International*, *2014*, 1–9. https://doi.org/10.1155/2014/395434

Rolfhamre, P., Grabowska, K., & Ekdahl, K. (2004). Implementing a public web based GIS service for feedback of surveillance data on communicable diseases in Sweden. *BMC Infectious Diseases*, *4*(1), 17. https://doi.org/10.1186/1471-2334-4-17

Saha, S. B., Uttam, V., & Verma, V. (2015). u-CARE: user-friendly Comprehensive Antibiotic resistance Repository of Escherichia coli. *Journal of Clinical Pathology*, *68*(8), 648–651. https://doi.org/10.1136/jclinpath-2015-202927

Sandmann, S., Schaumburg, F., & Varghese, J. (2023). GEFAAR: a generic framework for the analysis of antimicrobial resistance providing statistics and cluster analyses. *Scientific Reports*, *13*(1), 16922. https://doi.org/10.1038/s41598-023-44109-3

Schindler, J., Schindler, Z., & Schindler, J. (1998). A WWW-based information system on resistance of bacteria to antibiotics. *Medical Informatics*, *23*(3), 179–185. https://doi.org/10.3109/14639239809001396

Stedtfeld, R. D., Williams, M. R., Fakher, U., Johnson, T. A., Stedtfeld, T. M., Wang, F., Khalife, W. T., Hughes, M., Etchebarne, B. E., Tiedje, J. M., & Hashsham, S. A. (2016). Antimicrobial resistance dashboard application for mapping environmental occurrence and resistant pathogens. *FEMS Microbiology Ecology*, *92*(3), fiw020. https://doi.org/10.1093/femsec/fiw020

Stelling, J., & Brien, T. F. (2016). WHONET: Software for Surveillance of Infecting Microbes and Their Resistance to Antimicrobial Agents. In *Molecular Microbiology* (pp. 692–706). American Society of Microbiology. https://doi.org/10.1128/9781555819071.ch48

Steurbaut, K., Van Hoecke, S., Colpaert, K., Lamont, K., Taveirne, K., Depuydt, P., Benoit, D., Decruyenaere, J., & De Turck, F. (2010). Use of web services for computerized medical decision support, including infection control and antibiotic management, in the intensive care unit. *Journal of Telemedicine and Telecare*, *16*(1), 25–29. https://doi.org/10.1258/jtt.2009.001008

Teodoro, D., Pasche, E., Gobeill, J., Emonet, S., Ruch, P., & Lovis, C. (2012). Building a Transnational Biosurveillance Network Using Semantic Web Technologies: Requirements, Design, and Preliminary Evaluation. *Journal of Medical Internet Research*, *14*(3), e73. https://doi.org/10.2196/jmir.2043

Thursky, K. A., Buising, K. L., Bak, N., Macgregor, L., Street, A. C., Macintyre, C. R., Presneill, J. J., Cade, J. F., & Brown, G. V. (2006). Reduction of broad-spectrum antibiotic use with computerized decision support in an intensive care unit. *International Journal for Quality in Health Care*, *18*(3), 224–231. https://doi.org/10.1093/intqhc/mzi095

Thursky, K., Robertson, M., Luu, S., Black, J., Richards, M., & Buising, K. (2009). Guidance DS: A web-based clinical decision support system for antimicrobial stewardship in hospitals. *IFAC Proceedings Volumes*, *42*(12), 354–358. https://doi.org/10.3182/20090812-3-DK-2006.0104

Tsutsui, A., & Suzuki, S. (2018). Japan nosocomial infections surveillance (JANIS): a model of sustainable national antimicrobial resistance surveillance based on hospital diagnostic microbiology laboratories. *BMC Health Services Research*, *18*(1), 799. https://doi.org/10.1186/s12913-018-3604-x

Vatopoulos, A. C., Kalapothaki, V., & Legakis, N. J. (1999). An electronic network for the surveillance of antimicrobial resistance in bacterial nosocomial isolates in Greece. The Greek Network for the Surveillance of Antimicrobial Resistance. *Bulletin of the World Health Organization*, *77*(7), 595–601. http://www.ncbi.nlm.nih.gov/pubmed/10444883

Wallace, S., & Damani, N. (2013). O022: Development of an electronic dashboard to assist surveillance. *Antimicrobial Resistance and Infection Control*, *2*(S1), O22. https://doi.org/10.1186/2047-2994-2-S1-O22

Wang, M., Goh, Y.-X., Tai, C., Wang, H., Deng, Z., & Ou, H.-Y. (2022). VRprofile2: detection of antibiotic resistance-associated mobilome in bacterial pathogens. *Nucleic Acids Research*, *50*(W1), W768–W773. https://doi.org/10.1093/nar/gkac321

Yadav, S. K., Shrestha, L., Acharya, J., Gompo, T. R., Chapagain, S., & Jha, R. (2023). Integrative Digital Tools to Strengthen Data Management for Antimicrobial Resistance Surveillance in the “One Health” Domain in Nepal. *Tropical Medicine and Infectious Disease*, *8*(6), 291. https://doi.org/10.3390/tropicalmed8060291

Yin, X., Zheng, X., Li, L., Zhang, A.-N., Jiang, X.-T., & Zhang, T. (2023). ARGs-OAP v3.0: Antibiotic-Resistance Gene Database Curation and Analysis Pipeline Optimization. *Engineering*, *27*, 234–241. https://doi.org/10.1016/j.eng.2022.10.011
