# Supplementary material for: Characterizing approaches used to display antimicrobial resistance data in veterinary and human medicine: a scoping review
Source: Antimicrob Steward Healthc Epidemiol. 2025 Dec 17;5(1):e344. doi: 10.1017/ash.2025.10243 (PMC12722559; doi:10.1017/ash.2025.10243)
Supplement: Alberts et al. supplementary material [file S2732494X2510243Xsup001.zip › S4 Table.docx]

**S4 Table** First Author Country. The country affiliation of the first author.

| **Country** | **Number of Publications**  **(n = 42)*** | **Percentage (%)** |
| --- | --- | --- |
|  |  |  |
| United States of America | 5 | 11.9 |
| United Kingdom | 4 | 9.5 |
| Canada | 3 | 7.1 |
| China | 3 | 7.1 |
| Switzerland | 3 | 7.1 |
| Australia | 2 | 4.8 |
| India | 2 | 4.8 |
| Spain | 2 | 4.8 |
| Sweden | 2 | 4.8 |
| Austria | 1 | 2.4 |
| Belgum | 1 | 2.4 |
| Czech Republic | 1 | 2.4 |
| Finland | 1 | 2.4 |
| France | 1 | 2.4 |
| Germany | 1 | 2.4 |
| Greece | 1 | 2.4 |
| Iran | 1 | 2.4 |
| Japan | 1 | 2.4 |
| Nepal | 1 | 2.4 |
| Netherlands | 1 | 2.4 |
| Portugal | 1 | 2.4 |
| Romania | 1 | 2.4 |
| Slovakia | 1 | 2.4 |
| South Africa | 1 | 2.4 |
| Thailand | 1 | 2.4 |

* Some publications report multiple country affiliations.
