# Supplementary material for: Characterizing approaches used to display antimicrobial resistance data in veterinary and human medicine: a scoping review
Source: Antimicrob Steward Healthc Epidemiol. 2025 Dec 17;5(1):e344. doi: 10.1017/ash.2025.10243 (PMC12722559; doi:10.1017/ash.2025.10243)
Supplement: Alberts et al. supplementary material [file S2732494X2510243Xsup001.zip › S5 Table.docx]

**S5 Table** Database names of the data used for AMR displays.

| **Database Name** | **Number of Publications**  **(n = 42)*** | **Percentage (%)** |
| --- | --- | --- |
|  |  |  |
| Unspecific name(s) | 19 | 45.2 |
| Pubmed | 5 | 11.9 |
| GenBank | 4 | 9.5 |
| NCBI | 2 | 4.8 |
| AmSurv Database | 1 | 2.4 |
| Antimicrobial Resistance Database System | 1 | 2.4 |
| The Comprehensive Antibiotic Resistance Database (CARD) | 1 | 2.4 |
| Canadian Integrated Program for Antimicrobial Resistance Surveillance (CIPARS) | 1 | 2.4 |
| Computer-based Surveillance and Alerting of Nosocomial Infections, Antimicrobial Resistance and Antibiotic Consumption (COSARA) Database | 1 | 2.4 |
| Chemical Entities of Biological Interest (ChEBI) | 1 | 2.4 |
| Division Informatique de L’Hôpital Cantonal de Genève (DIOGENE) | 1 | 2.4 |
| EpiArk | 1 | 2.4 |
| European Nucleotide Archive | 1 | 2.4 |
| Google | 1 | 2.4 |
| ICEberg | 1 | 2.4 |
| ICMR's Antimicrobial ResistanceSurveillance system (i-AMRSS) | 1 | 2.4 |
| Infectious Diseases Surveillance Information System for Antimicrobial Resistantce (ISIS-AR) | 1 | 2.4 |
| InfoMed | 1 | 2.4 |
| Iranian Antimicrobial Resistance (AMR) Surveillance System | 1 | 2.4 |
| Japan Nosocomial Infections Surveillance (JANIS) | 1 | 2.4 |
| Kyoto Encyclopedia of Genes and Genomes (KEGG) DRUG | 1 | 2.4 |
| Laboratory Information System (LIS) | 1 | 2.4 |
| NHS AMRCQUIN trust data | 1 | 2.4 |
| NHS Digital | 1 | 2.4 |
| NHS healthcare worker surveillance data | 1 | 2.4 |
| Pathogenicity Island Database (PAIDB) | 1 | 2.4 |
| PHAge Search Tool Enhanced Release (PHASTER) | 1 | 2.4 |
| Public Health England (PHE) lab information management systems | 1 | 2.4 |
| PLSDB | 1 | 2.4 |
| PhD and Masters theses and conference proceedings from selected veteriary schools | 1 | 2.4 |
| PubChem | 1 | 2.4 |
| Resistance Open | 1 | 2.4 |
| Royal Melbourne Hospital Data | 1 | 2.4 |
| Scopus | 1 | 2.4 |
| Swiss-Prot | 1 | 2.4 |
| The MicrobDynamic System | 1 | 2.4 |
| TnRegistry (The Transposons Registry) | 1 | 2.4 |
| Web of Science | 1 | 2.4 |
| An Admit-Discharge-Transfer (ADT) system | 1 | 2.4 |
| A Hospital Census System | 1 | 2.4 |
| * Publications may use multiple databases. |  |  |
