# Supplementary material for: Characterizing approaches used to display antimicrobial resistance data in veterinary and human medicine: a scoping review
Source: Antimicrob Steward Healthc Epidemiol. 2025 Dec 17;5(1):e344. doi: 10.1017/ash.2025.10243 (PMC12722559; doi:10.1017/ash.2025.10243)
Supplement: Alberts et al. supplementary material [file S2732494X2510243Xsup001.zip › S6 Table.docx]

**S6 Table** Bacteria genera used for AMR displays.

| **Genus** | **Number of Publications**  **(n = 42)*** | **Percentage (%)** |
| --- | --- | --- |
|  |  |  |
| *Enterococcus* | 2 | 4.8 |
| *Escherichia* | 2 | 4.8 |
| *Salmonella* | 2 | 4.8 |
| *Staphylococcus* | 2 | 4.8 |
| *Acinetobacter* | 1 | 2.4 |
| *Campylobacter* | 1 | 2.4 |
| *Citrobacter* | 1 | 2.4 |
| *Clostridium* | 1 | 2.4 |
| *Enterobacter* | 1 | 2.4 |
| *Klebsiella* | 1 | 2.4 |
| *Pseudomonas* | 1 | 2.4 |
|  |  |  |

* Publications may use multiple genera.
