# Supplementary material for: Characterizing approaches used to display antimicrobial resistance data in veterinary and human medicine: a scoping review
Source: Antimicrob Steward Healthc Epidemiol. 2025 Dec 17;5(1):e344. doi: 10.1017/ash.2025.10243 (PMC12722559; doi:10.1017/ash.2025.10243)
Supplement: Alberts et al. supplementary material [file S2732494X2510243Xsup001.zip › S7 Table.docx]

**S7 Table** Bacteria species used for AMR displays.

| **Species** | **Number of Publications**  **(n = 42)*** | **Percentage (%)** |
| --- | --- | --- |
|  |  |  |
| *Escherichia coli (E. coli)* | 7 | 16.7 |
| *Staphylococcus aureus* | 7 | 16.7 |
| *Klebsiella pneumoniae* | 4 | 9.5 |
| *Pseudomonas aeruginosa* | 4 | 9.5 |
| *Streptococcus pneumoniae* | 4 | 9.5 |
| *Enterococcus faecalis* | 2 | 4.8 |
| *Acinetobacter baumannii* | 1 | 2.4 |
| *Acinetobacter spp.* | 1 | 2.4 |
| *Campylobacter spp.* | 1 | 2.4 |
| *Citrobacter koseri* | 1 | 2.4 |
| *Clostridium difficile* | 1 | 2.4 |
| *Coagulase-negative Staphylococci (CoNS)* | 1 | 2.4 |
| *Enterobacter cloacae* | 1 | 2.4 |
| *Enterobacter spp.* | 1 | 2.4 |
| *Enterococcus faecium* | 1 | 2.4 |
| *Streptococcus pyogenes -Group A Streptococci* | 1 | 2.4 |
| *Streptococcus agalactiae -Group B Streptococci* | 1 | 2.4 |
| *Klebsiella oxytoca* | 1 | 2.4 |
| *Morganella morganii* | 1 | 2.4 |
| *Proteus mirabilis* | 1 | 2.4 |
| *Salmonella enterica serovar Typhimurium* | 1 | 2.4 |
| *Salmonella spp.* | 1 | 2.4 |
| *Staphylococcus saprophyticus* | 1 | 2.4 |
| *non-typhoidal Salmonella spp.* | 1 | 2.4 |
|  |  |  |

* Publications may use multiple species.
