# Supplementary material for: Characterizing approaches used to display antimicrobial resistance data in veterinary and human medicine: a scoping review
Source: Antimicrob Steward Healthc Epidemiol. 2025 Dec 17;5(1):e344. doi: 10.1017/ash.2025.10243 (PMC12722559; doi:10.1017/ash.2025.10243)
Supplement: Alberts et al. supplementary material [file S2732494X2510243Xsup001.zip › S8 Table.docx]

**S8 Table** Sample types used in displays with sample restrictions.

| **Sample type** | **Number of Publications**  **(n = 42)*** | **Percentage (%)** |
| --- | --- | --- |
|  |  |  |
| Blood | 5 | 11.9 |
| Urine | 5 | 11.9 |
| Respiratory | 3 | 7.1 |
| Genital | 1 | 2.4 |
| Non-sterile | 1 | 2.4 |
| Other sterile | 1 | 2.4 |
| Pus/Wound | 1 | 2.4 |
| Stool | 1 | 2.4 |
| Stool | 1 | 2.4 |
| Surgical site samples | 1 | 2.4 |
| Wastewater | 1 | 2.4 |
| Not Stated | 1 | 2.4 |
