# Supplementary material for: Characterizing approaches used to display antimicrobial resistance data in veterinary and human medicine: a scoping review
Source: Antimicrob Steward Healthc Epidemiol. 2025 Dec 17;5(1):e344. doi: 10.1017/ash.2025.10243 (PMC12722559; doi:10.1017/ash.2025.10243)
Supplement: Alberts et al. supplementary material [file S2732494X2510243Xsup001.zip › S9 Table.docx]

**S9 Table** Intended users and the categorization.

| **User** | **Category** | **Number of Publications**  **(n = 42)*** | **Percentage (%)** |
| --- | --- | --- | --- |
|  |  |  |  |
|  |  |  |  |
| Primary care organizations | Health Professionals | 28 | 66.7 |
| Researchers | Researchers | 18 | 42.9 |
| Public Health and Policy-makers | Policy Makers | 14 | 33.3 |
| General Public | Public | 7 | 16.7 |
| Pharmacists | Health Professionals | 4 | 9.5 |
| Epidemiologists | Researchers | 2 | 4.8 |
| Laboratories | Researchers | 2 | 4.8 |
| Not stated | Not Stated | 2 | 4.8 |
| Breeding Enterprises | Researchers | 1 | 2.4 |
| Drug manufacturers | Researchers | 1 | 2.4 |
| Engineers | Researchers | 1 | 2.4 |
| Field officers | Policy Makers | 1 | 2.4 |
| Hospital infection control managers | Health Professionals | 1 | 2.4 |
| Infectionists | Researchers | 1 | 2.4 |
| Microbiologists | Researchers | 1 | 2.4 |
| Monitoring institutions | Policy Makers | 1 | 2.4 |
| Public health risk managers | Policy Makers | 1 | 2.4 |
| Research funders | Researchers | 1 | 2.4 |
